# Engagement in care among women and their infants lost to follow-up under Option B+ in eSwatini

**DOI:** 10.1371/journal.pone.0222959

**Published:** 2019-10-30

**Authors:** William Reidy, Harriet Nuwagaba-Biribonwoha, Siphesihle Shongwe, Ruben Sahabo, Kieran Hartsough, Yingfeng Wu, Averie Gachuhi, Simangele Mthethwa-Hleta, Elaine J. Abrams

**Affiliations:** 1 ICAP at Columbia, Mailman School of Public Health, Columbia University, New York, New York, United States of America; 2 Department of Epidemiology, Columbia University, Mailman School of Public Health, New York, New York, United States of America; 3 Ministry of Health, Mbabane, Eswatini; 4 Department of Pediatrics, Vagelos College of Physicians & Surgeons, Columbia University, New York, New York, United States of America; University of Washington, UNITED STATES

## Abstract

**Background:**

Universal antiretroviral treatment (ART) for pregnant women with HIV, Option B+, has been adopted widely for prevention of mother-to-child HIV transmission (PMTCT). Some evidence shows high loss to follow-up (LTF) under this model. However, gaps in data systems limit this evidence. We collected additional information for women and infants LTF from Option B+ in Eswatini to assess more accurate outcomes.

**Methods:**

LTF at 6-months postpartum was assessed using facility data. Additional data was gathered from: 1) the national ART database and paper records; 2) patient tracing; and 3) interviews and abstraction from patient-held records. Engagement in care was defined as any clinic visit within 91 days before or after 6-months postpartum or completion of a documented transfer; or, for those traced but not completing study interviews, visits at 6-months postpartum or later (for infants), or visits within 3-months of tracing (for women). Multivariable loglinear models were used to identify correlates of engagement.

**Results:**

One-hundred-ninety-four (44.7%) of 434 LTF women had outcomes ascertained, including 122 (62.9%) women engaged in care. Among 510 LTF infants, 265 (52.0%) had ascertained outcomes, including 143 (54.0%) engaged in care, 47 (17.7%) pregnancy losses, and 18 (6.8%) deaths. Seventy-two of 189 live infants (38.1%) with ascertained outcomes had a 6-week early infant diagnostic (EID) test. Among women with ascertained outcomes, gestational age of 20+ weeks (vs. fewer than 20 weeks, aRR 0.80; 95% CI 0.68–0.94) and age 25–29 years (vs. 15–24 years, aRR 0.81; 95% CI 0.67–0.97), were associated with lower engagement; initiating ART after first ANC visit was associated with higher engagement (vs. at first ANC visit, aRR 1.12; 95% CI 1.04–1.21). Among infants with ascertained outcomes, mother not initiating ART was associated with lower engagement (vs. ART at first ANC visit, aRR 0.71; 95% CI 0.54–0.91).

**Conclusion:**

Substantial numbers of women and infants classified as LTF under Option B+ were engaged in care, though a suboptimal level of 6-week EID testing was observed. These findings highlight a need to improve coverage of routine EID testing, and improve data systems to better capture PMTCT patient outcomes.

## Introduction

Implementation of universal antiretroviral treatment (ART) for prevention of mother-to-child transmission (PMTCT), known widely as Option B+, has led to a rapid increase in ART coverage among pregnant women living with HIV in many countries in sub-Saharan Africa.[1–3] However, concerns about patient retention under this model arose during early implementation, and subsequent evidence has not assuaged those concerns.[4–9] Research to date has shown heterogeneous and oftentimes high levels of loss to follow-up among women receiving Option B+ services.[7–9] While fewer studies have examined retention among infants under Option B+, evidence available shows substantial proportions of infants not receiving essential PMTCT services.[10,11]

Nearly all of these findings, however, are limited by a reliance on programmatic data systems that do not effectively track women and infants across services and health facilities.[7] This is reflected in the use of LTF, rather than engagement in care, as the primary measure of retention. Much previous research among patients classified as LTF from general ART services found that many had, in fact, engaged in care in a different health facility, or died.[12–15] Such research is far less common among recipients of PMTCT care, in particular under Option B+.[16–18]

The Kingdom of Eswatini has among highest HIV prevalence and incidence, of any country worldwide, with nearly 40% of women receiving antenatal services diagnosed with HIV infection.[19–21] In 2013–15, an implementation science study was conducted with the Eswatini Ministry of Health (MOH) to assess ART uptake and retention among pregnant and postpartum women at health facilities transitioning to Option B+.[8] Study data was abstracted from routinely-collected health records within each facility. A preliminary analysis of study data showed high LTF among the 1,221 mother-infant pairs in the Option B+ study arm—with more than one-third of women and infants, respectively, LTF by 6 months postpartum.[22] Recognizing the limitations of programmatic data in measuring engagement in care, we aimed to collect additional information on these LTF women and infants to obtain accurate estimates of engagement in care under Option B+ and assess correlates of engagement among this population initially classified as LTF.

## Materials and methods

### Study design

This study assessed outcomes among a population of women living with HIV who had initiated PMTCT services under Option B+ between September 2013-August 2014 at 10 facilities in Eswatini, who had previously not received ART, and were classified as LTF from services by 6 months after delivery. Additional details on LTF classification are provided below. This population initiated antenatal care as an implementation science study utilizing a stepped-wedge design was underway in these facilities; under this study, facilities transitioned from PMTCT Option A (the national standard of care at the time) to Option B+ for women living with HIV initiating PMTCT services and data on mother and infant retention was collected through 6 months postpartum.[8] Activities under the subsequent study to determine outcomes among patients LTF by 6 months after delivery, which is the focus of this paper, were conducted between March 2016-May 2017.

### Setting

The selection of facilities included in this study, the transition to Option B+ within these facilities, and other relevant information about the prior evaluation were described previously.[8] Facilities transitioned to Option B+ included one hospital, 3 public health centers, and 6 primary care clinics, located in urban (n = 6), semi-urban (n = 1) and rural (n = 3) settings. In all facilities, ART medical care was provided for pregnant and postpartum women in an integrated fashion within maternal and child health clinics. PMTCT services for infants, including early infant diagnostic (EID) HIV testing, were provided in immunization clinics located within the facilities. All 10 facilities provided patient tracing services conducted by telephone by facility staff; in addition, community-based tracing was provided for patients of all 10 facilities, though responsibility for provision of these services differed by facility (see S1 Table) and many patient outcomes ascertained during tracing were historically not documented in a standardized, structured way. All facilities offered support for ART adherence and retention to HIV-positive pregnant through a peer mentor program.

### Participants and procedures

Women and infants classified as LTF from Option B+ services at study facilities, based on a preliminary analysis of data from the prior evaluation, were included in this study. More specifically, all women with a first ANC visit at a study facility following implementation of Option B+ and who were not engaged in services through 6 months after delivery, defined as no clinical visit within 91 days of 6 months postpartum, were included. Clinical visits included, for women, ART visits documented in the patient ART medical record and, for infants, visits documented in the facility immunization clinic register. Women already receiving ART were not eligible for inclusion in the prior evaluation, and are not included in this study. For purposes of defining the infant study population, all women receiving Option B+ services were assumed to have potentially delivered a live infant. Women not linked via clinic records to infants found to be engaged in immunization clinic services through 6 months postpartum (based on a documented or estimated delivery date), were deemed to have LTF infants, and these LTF infants were included in the study.

This study sought through three phases to evaluate engagement in care among women and infants initially classified as LTF. The first study phase consisted of a review of existing paper-based and electronic records and abstraction of data relevant to our assessment of engagement in care. Paper-based records reviewed by the study team included MOH tools such as patient ART medical records and registers, ANC registers, child immunization clinic registers, and appointment registers, as well as non-MOH notebooks and registers maintained by peer mentors. Information from structured fields within these records was abstracted. In addition, information recorded more informally, such as notes in open text fields or in margins, was abstracted wherever it recorded pregnancy loss or maternal or infant death for study subjects—as these outcomes were largely not captured in structured fashion on MOH tools. Lastly, any notes indicating that the woman or infant had relocated outside of Eswatini were documented in study abstraction. Women and infants found in the data abstraction phase to have been engaged in care at 6 months postpartum; formally transferred to another facility or relocated outside of Eswatini; who had died; or, for subjects classified as LTF infants that were determined to have been pregnancy losses, a final outcome was considered to have been ascertained and no further study data collection activities were conducted.

All women and infants without a documented outcome from data abstraction, as described above, were eligible for the second study phase, patient tracing. Contact information from ART medical records for women were used for tracing. For women and infants eligible for tracing and with telephone contact information available, tracing via telephone was conducted by facility healthcare workers. For patients not reached who named and provided contact information for a treatment supporter, tracing calls were made to the treatment supporter. Patients not reached by phone after 4 attempts and not identified as having died by a treatment supporter were physically traced by individuals routinely providing community tracing for the health facilities, with up to 2 physical tracing attempts made. For tracing contacts successfully reached, telephone and community tracers conducted brief interviews to assess key information such as current HIV care and ART engagement and vital status of the woman and infant, where applicable, and recorded responses using a structured tracing form.

Women who were reached and completed the telephone or community tracing interview and who did not provide a definitive outcome—specifically, pregnancy loss or infant death for cases where the subject was a LTF infant—were introduced to study interviewers, who conducted informed consent procedures and, for consenting women, arranged a time for a study interview in a location convenient for the woman. Consenting women completed the third phase of the study, an in-person interview designed to capture information such as engagement in care and ART use since the time of LTF, reasons for disengaging from the study clinic, and infant HIV testing and infection status, as applicable. In addition, women were asked to provide patient-held national ART cards and/or infant immunization cards if available, in which case information on ART visits and infant clinic visits, including HIV testing, was abstracted.

### Outcomes

The primary outcome was maternal and/or infant engagement in care, defined as evidence of a clinic visit within 91 days (before or after) of 6-months postpartum. Sources of this evidence included clinic or patient-held records, the national ART database, and self-reported visit dates provided in study interviews. In addition, women who were found in clinic records or in the national ART database to have completed a formal transfer-out process (and were therefore not traced), or who reported current engagement in care in the tracing phase—defined as any clinic visit in the 3 months prior to tracing—and did not complete a study interview were classified as engaged in care. Similarly, for infants who were reported to be alive through tracing of mothers who did not subsequently complete study interviews, a report of a last visit to the immunization clinic within 91 days prior to 6-months postpartum, or at any point later—routine immunization visits were expected through 18 months of age—was considered evidence of engagement in care. Use of ART by women was collected through self-report in tracing and study interviews, and through abstraction of facility and patient-held records and the national ART database. Specifically, in the tracing interview, women were asked to identify the date when they most recently took ART; in the study interview, women were asked if they had taken ART in the past 3 months, and were asked to provide approximate first and last visit dates of attendance at each ART clinic ever visited as a patient. ART data collected from facility- and patient-held records and the national ART database consisted of ART clinic visit dates and dates of any patient outcomes recorded, such as death or LTF.

### Other outcomes among women

Other outcomes for women included death and having relocated outside of Eswatini, per data abstraction or tracing of a treatment supporter. Lastly for women, disengagement from care was defined among women completing a study interview as no clinic visit within 91 days of 6 months postpartum identified through data abstraction, per self-report or patient-held record; and, among women reached via tracing but not completing a study interview, as no clinic visit within 91 days of 6 months postpartum identified through data abstraction and no self-reported HIV care or ART visit in the past 3 months prior to tracing. Women without an outcome identified in data abstraction and not reached via tracing were classified as having unknown outcomes.

### Other infant outcomes

Other infant outcomes include those from prior to or at delivery, including pregnancy loss, death at delivery, transfer out of mother, and maternal relocation outside of Eswatini. With the exception of maternal transfer-out, which was documented formally in the patient ART medical record and/or national ART database, these outcomes could be assessed from a range of clinic-based records abstracted or from patient tracing. Other outcomes from after delivery include death, as identified from any abstraction or as reported by a mother or mother’s treatment supporter, disengagement from care, and 6-week EID testing, which was assessed from patient immunization records abstracted during study interviews or, if not available, through self-report by women.

### Statistical analysis

Data were analyzed using SAS 9.4 (SAS Institute, Cary, NC). Participants and health facility characteristics were summarized using proportions or medians with interquartile ranges (IQR); crude comparisons across sub-populations employed chi-square and Wilcoxon Rank Sum tests. Engagement in care and other outcomes for women was examined in bivariate analyses among the subpopulation of women for whom an outcome could be ascertained, as well as among the overall population of LTF women. Outcomes among cases initially classified as LTF infants, but without any documented evidence of a live infant subsequently collected—including pregnancy loss, death at delivery, and maternal transfer or relocation—were assessed among the full population of LTF infants. Outcomes among documented live infants—including infant engagement in care, death after delivery, and disengagement—were examined among this subset of documented live infants, and well as this group plus the additional infants who did not have an outcome ascertained under the study.

Loglinear regression was used to identify factors associated with engagement in care, including facility setting, maternal age group, gestational age at first ANC visit (< 20 weeks versus 20 or more weeks), knowledge of HIV status at first ANC visit, WHO stage at HIV care enrollment, and timing of ART initiation. In these models, engagement was in separate models among women and infants measured in relation to: 1) disengagement, death, or, for women, relocation out of the country; and 2) these same outcomes, as well as having an unknown outcome under the study. Generalized estimating equations were used to account for correlations between participants within health facilities and to calculate robust standard errors, and to calculate crude and adjusted risk ratios to estimate the associations between these factors and engagement in care. In addition, exploratory analyses were conducted to assess relationships between maternal and infant engagement in care, as well as pregnancy loss and infant death at delivery on maternal engagement in care. These were conducted as exploratory analyses given the substantial numbers of women and infants missing these outcomes, and the likely correlation between missing outcomes for mother and infant.

### Ethical approval

Ethical approval was provided by the Columbia University Medical Center Institutional Review Board and the Eswatini Scientific and Ethics Committee. The requirement for informed consent from individuals was waived for abstraction of routinely collected data, including data from patient tracing; mothers provided written consent for participation in the study interview.

## Results

### Primary outcomes among women and infants

**Fig 1:**
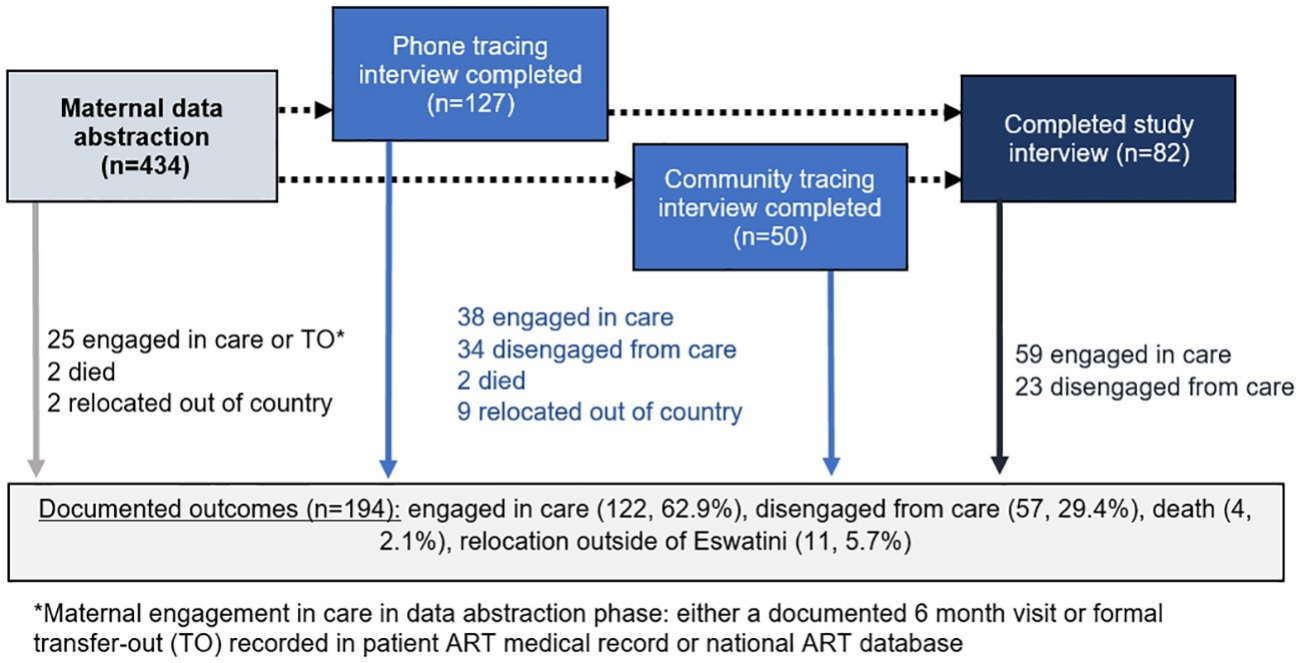
Outcomes from data abstraction, patient tracing, and study interviews for women.

Four hundred and thirty-four women and 510 infants classified as LTF from Option B+ services at study facilities were included in this study. Incorporating data collected across the study phases, 194 (44.7%) of the 434 LTF women had outcomes ascertained (Fig 1). Among those with outcomes ascertained, 122 (62.9%) were engaged in care at 6 months postpartum, 57 (29.4%) were disengaged from care, 11 (5.7%) had moved out of the country, and 4 (2.1%) had died. Information on ART engagement was available for 111 of the 122 (91%) women found to be engaged in care; among these women, 97 (87%) were receiving ART.

**Fig 2:**
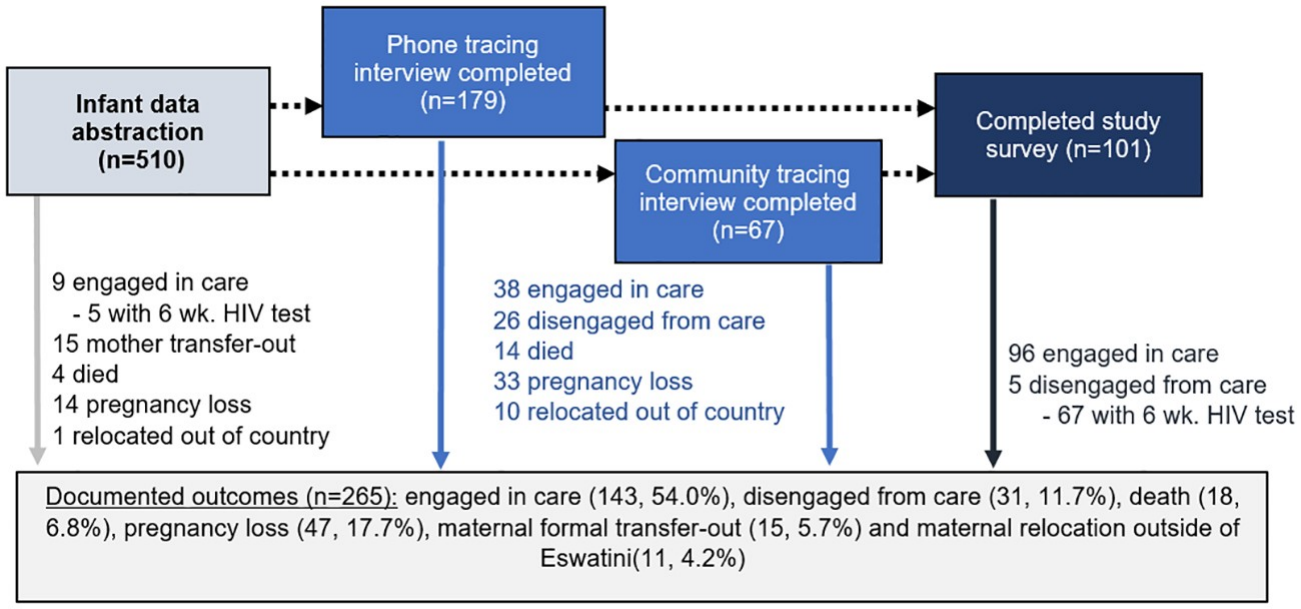
Outcomes from data abstraction, patient tracing, and study interviews for infants.

Among the 510 cases classified as LTF infants, 265 (52.0%) had an outcome determined (Fig 2). Seventy-six (14.9%) of these cases with outcomes, however, did not document a live infant—this included 47 (9.2%) pregnancy losses, 3 (0.6%) infants who died at delivery, 15 (2.9%) cases whether mothers were documented as transferring facilities, 11 (2.2%) mothers who moved out of Eswatini and could not be plausibly traced. Of the remaining 434 LTF infants—excluding the 76 cases with outcomes precluding the tracing of a live infant—189 (43.5%) live infants had outcomes ascertained, including 143 (75.7% of 189 documented live infants with known outcomes) who were engaged at clinics, 31 (16.4%) disengaged from care, and 15 (7.9%) who subsequently died by 6 months after delivery. Altogether, 72 of these 189 infants (38.1%) were determined to have had an EID test at 6 weeks.

### Ascertainment of outcomes across study phases

As summarized in Figs 1 and 2, among the 434 pregnant women and 510 infants initially classified as LTF at 6 months postpartum, 29 (7%) women and 43 (8%) infants had final outcomes determined through the initial data abstraction phase; 177 women (44% of 405 remaining women) and 246 infants (53% of 467 remaining infants) were successfully traced by phone or in the community and 11 women and 57 infants, respectively, had outcomes determined within this phase—death, pregnancy loss, relocation outside of Eswatini—that precluded recruitment for a study interview. Among women traced, 49% (82) of 166 women eligible for study survey recruitment consented and completed a study interview, including 36 (44% of 82) who provided a patient-held ART card for abstraction; in addition, mothers of 101 of the 189 eligible LTF infants (53%) completed study interviews, and 71 (69%) of these 101 women provided infant immunization cards for abstraction.

### Factors related to successful mother and infant tracing

Maternal and facility characteristics for women and infants LTF, eligible for, and reached via tracing are summarized in Table 1. The one factor related to successful tracing of women LTF in bivariate analyses (p<0.05) was the time between HIV care enrolment and tracing; this factor, along with mother’s age, and timing of mother’s ART initiation were associated with successful tracing of LTF infants as well. Specifically, women and infants who were successfully traced each had a lower median duration between HIV care enrollment and tracing than the populations not reached via tracing. Additionally, smaller proportions of infants traced (8%), as compared to the overall population eligible for tracing (13%), had never started ART and mothers of infants successfully traced were slightly older.

**Table 1:** Facility and maternal characteristics at first ANC visit or HIV care enrollment among women and infants eligible for tracing, among those found via patient tracing, and enrolled in the study survey.

	Women				Infants			
	All LTF women	Eligible for tracing	Eligible for tracing, were not traced	Reached via tracing	All LTF infants	Eligible for tracing	Eligible for tracing, were not traced	Reached via tracing
	(n = 434)	(n = 405)	(n = 228)	(n = 177)	(n = 510)	(n = 464)	(n = 218)	(n = 246)
	N (%)	N (%)	N (%)	N (%)	N (%)	N (%)	N (%)	N (%)
Age group (years)								
Median (IQR)	24 (22–28)	24 (22–28)	24 (22–27)	25 (22–28)	25 (22–29)	25 (22–28)	25 (22–28)	**25 (23–30)**
15–24	211 (49)	202 (50)	117 (51)	85 (47)	228 (45)	210 (45)	104 (48)	**106 (43)**
25–29	135 (31)	125 (31)	72 (32)	53 (30)	159 (31)	146 (31)	73 (33)	**73 (30)**
≥30	76 (18)	66 (16)	32 (14)	34 (19)	111 (22)	98 (21)	35 (16)	**63 (26)**
*Missing*	*12 (3)*	*12 (3)*	*7 (3)*	*5 (3)*	*12 (2)*	*10 (2)*	*6 (3)*	***4 (2)***
Gestational age (weeks)								
Median (IQR)*	20 (16–25)	20 (16–25)	20 (16–24)	20 (16–26)	19 (15–24)	20 (15–25)	20 (16–24)	19 (14–25)
HIV Status at ANC enrollment								
Known positive	192 (44)	178 (44)	97 (43)	81 (46)	232 (45)	211 (45)	94 (43)	117 (48)
New positive	242 (56)	227 (56)	131 (57)	96 (54)	278 (55)	253 (55)	124 (57)	129 (52)
Timing of ART initiation								
At first ANC visit	327 (75)	304 (75)	168 (74)	136 (77)	407 (80)	369 (80)	164 (75)	**205 (83)**
After first ANC visit	30 (7)	26 (6)	14 (6)	12 (7)	40 (8)	37 (8)	16 (7)	**21 (9)**
Never received ART	77 (18)	75 (19)	46 (20)	29 (16)	63 (12)	58 (13)	38 (17)	**20 (8)**
Time from first HIV care visit to first tracing attempt (in days)								
Median (IQR)	-	904 (822–979)	923 (841–982)	**889 (805–955)**	-	892 (812–971)	919 (841–982)	**864 (793–951)**

Chi square p<0.05 in bold. Facility setting (urban, semi-urban, rural) and maternal WHO stage and CD4 cell count were also examined across groups, and no differences were observed.

### Factors related to engagement in care among all LTF subjects and subjects with known outcomes

Table 2 presents frequencies of ascertained outcomes—engaged in care, disengaged, dead, or moved out of Eswatini—by maternal and facility characteristics among LTF women. Proportions are presented separately for each outcome among the full population of 434 LTF women, and among the subset of 194 women with ascertained outcomes. When examining known outcomes among the population of 434 LTF women, engagement in care did not differ across any characteristics. Among the 194 women with known outcomes, engagement was associated with gestational age at first ANC visit (68.3% engaged among < 20 weeks, 53.3% among 20+ weeks). Additionally, in exploratory analyses, engagement in care was somewhat higher among women with known outcomes who had pregnancy loss or infant death at delivery (76.5% vs. 61.6%, S2 Table). Engagement in care was significantly higher among all LTF women with pregnancy loss or infant death at delivery (50.0% vs. 26.7%, S3 Table), though this in part was driven by the large number of cases where both mom and baby had unascertained outcomes (S4 Table).

**Table 2:** Outcomes among LTF women by facility and maternal characteristics at first ANC visit or HIV care enrollment.

	Engaged in care^1^			Disengaged from care			Died			Moved out of the country		All known outcomes	Unknown outcome		Total LTF women
	N	% of all LTF	*% among women with known outcomes*	n	% of all LTF	*% among women with known outcomes*	n	% of all LTF	*% among women with known outcomes*	n	% of all LTF	% of all LTF	n	% of all LTF	n
	**122**	**28.1%**	***62*.*9%***	**57**	**13.1%**	***29*.*4%***	**4**	**0.9%**	***2*.*1%***	**11**	**2.5%**	**44.7%**	**240**	**55.3%**	**434**
Age group (years)															
15–24	56	26.5%	*66*.*7%*	22	10.4%	*26*.*2%*	1	0.5%	*1*.*2%*	5	2.4%	39.8%	127	60.2%	211
25–29	34	25.2%	*56*.*7%*	20	14.8%	*33*.*3%*	1	0.7%	*1*.*7%*	5	3.7%	44.4%	75	55.6%	135
≥30	26	34.2%	*59*.*1%*	15	19.7%	*34*.*1%*	2	2.6%	*4*.*5%*	1	1.3%	57.9%	32	42.1%	76
*Missing*	6	50.0%	*100*.*0%*	0	0.0%	*0*.*0%*	0	0.0%	*0*.*0%*	0	0.0%	50.0%	6	50.0%	12
Gestational age (weeks)^2^															
<20	**72**	31.4%	***69*.*2%***	26	11.4%	*25*.*0%*	3	1.3%	*2*.*9%*	3	1.3%	45.4%	125	54.6%	229
20 or more	**50**	24.4%	***55*.*6%***	31	15.1%	*34*.*4%*	1	0.5%	*1*.*1%*	8	3.9%	43.9%	115	56.1%	205
HIV Status at 1st ANC															
Known positive	54	28.1%	*60*.*0%*	28	14.6%	*31*.*1%*	2	1.0%	*2*.*2%*	6	3.1%	46.9%	102	53.1%	192
New positive	68	28.1%	*65*.*4%*	29	12.0%	*27*.*9%*	2	0.8%	*1*.*9%*	5	2.1%	43.0%	138	57.0%	242
Timing of ART initiation															
At first ANC visit	96	29.4%	*63*.*6%*	43	13.1%	*28*.*5%*	4	1.2%	*2*.*6%*	8	2.4%	46.2%	176	53.8%	327
After first ANC visit	10	33.3%	*66*.*7%*	3	10.0%	*20*.*0%*	0	0.0%	*0*.*0%*	2	6.7%	50.0%	15	50.0%	30
Never received ART	16	20.8%	*57*.*1%*	11	14.3%	*39*.*3%*	0	0.0%	*0*.*0%*	1	1.3%	36.4%	49	63.6%	77

^1^Engagement among women includes documented transfers to other facilities

^2^GA cutoff based on median value (19 weeks)

Disengaged from care outcomes p<0.05 per chi square in bold. Facility setting (urban, semi-urban, rural) and maternal WHO stage and CD4 cell count were also examined across groups, and no differences were observed

Tables 3 and 4 summarize outcomes for LTF infants, including outcomes among cases with no documented evidence of a live infant (Table 3), as well as outcomes among documented live infants (Table 4). For the latter group of outcomes, proportions among the 434 live infants with documented outcomes (n = 189) plus infants with unknown outcomes under this study (n = 245), as well as proportions among the 189 live infants with documented outcomes are presented. Engagement in care, when examined among the combined group of live infants and infants with unknown outcomes, differed by timing of maternal ART initiation, with 35.9% of infants of women who had started ART at the first ANC visit engaged in care at 6 months postpartum, versus 23.3% who started ART after the first ANC visit and 19.6% who never started ART engaged in care. Limited to the subgroup of 189 live infants with known outcomes, no characteristics were clearly associated with engagement in care. Results of exploratory analyses showed slightly higher infant engagement among women who were also engaged in care (78.4%) compared to engagement among women documented as disengaged (71.1%) (S5 Table) or either disengaged or having an unknown outcome (65.9%) (S6 Table). Further, among 87 infants with adequate information from study interviews and data abstraction, infant 6-week EID testing was higher among women found to be engaged in care (86.1%) than among women disengaged from care (66.7%) (S7 Table).

**Table 3:** Outcomes among LTF infants by facility and maternal characteristics at first ANC visit or HIV care enrollment.

	Total LTF infants	No documented live infant									
		Pregnancy loss		Mom transferred out		Mom moved out of country		Died at delivery		Subtotal	
	n	n	% of all LTF	n	% of all LTF	n	% of all LTF	n	% of all LTF	n	% of all LTF
	**510**	**47**	**9.2%**	**15**	**2.9%**	**11**	**2.5%**	**3**	**0.6%**	**76**	**14.9%**
Age group (years)										0	
15–24	228	20	8.8%	8	3.5%	7	3.1%	0	0.0%	35	15.4%
25–29	159	16	10.1%	3	1.9%	2	1.3%	1	0.6%	22	13.8%
≥30	111	10	9.0%	4	3.6%	2	1.8%	2	1.8%	18	16.2%
*Missing*	12	1	8.3%	0	0.0%	0	0.0%	0	0.0%	1	8.3%
Gestational age (weeks)^1^											
<20	276	39	14.1%	12	4.3%	1	0.4%	1	0.4%	53	19.2%
20 or more	234	8	3.4%	3	1.3%	10	4.3%	2	0.9%	23	9.8%
HIV Status at ANC enrollment											
Known positive	232	23	9.9%	6	2.6%	7	3.0%	2	0.9%	38	16.4%
New positive	278	24	8.6%	9	3.2%	4	1.4%	1	0.4%	38	13.7%
ART initiation per clinic records											
At first ANC visit	407	36	8.8%	8	2.0%	8	2.0%	2	0.5%	54	13.3%
After first ANC visit	40	7	17.5%	1	2.5%	1	2.5%	1	2.5%	10	25.0%
Never received ART	63	4	6.3%	2	3.2%	2	3.2%	0	0.0%	8	12.7%

^1^GA cutoff based on median value (19 weeks)

^2^Excludes 76 cases of documented pregnancy loss, death at delivery, or maternal transfer out or relocation out of country from the original population of 510 L:TF infants.

Engaged from care outcomes p<0.05 per chi square in bold. Facility setting (urban, semi-urban, rural) and maternal WHO stage and CD4 cell count were also examined across groups, and no differences were observed.

**Table 4:** Outcomes among LTF infants by facility and maternal characteristics at first ANC visit or HIV care enrollment.

	Outcomes among live infants									Outcomes among all LTF infants				TOTAL	
	Engaged in care			Disengaged from care			Died after delivery			All known outcomes		Unknown outcome		Total live infants + unknown outcomes^2^	Total LTF infants
	n	% of all live + unknown	*% among live infants with outcomes*	n	% of all live + unknown	*% among live infants with outcomes*	n	% of all live + unknown	*% among live infants with outcomes*	n	% of all LTF	n	% of all LTF	n	n
	**143**	**32.9%**	***75*.*7%***	**31**	**7.1%**	***16*.*4%***	**15**	**3.5%**	***7*.*9%***	**265**	**52.0%**	**245**	**48.0%**	**434**	**510**
Age group (years)										0					
15–24	57	29.5%	*80*.*3%*	10	5.2%	*14*.*1%*	4	2.1%	*5*.*6%*	106	46.5%	122	53.5%	193	228
25–29	46	33.6%	*78*.*0%*	8	5.8%	*13*.*6%*	5	3.6%	*8*.*5%*	81	50.9%	78	49.1%	137	159
≥30	35	37.6%	*64*.*8%*	13	14.0%	*24*.*1%*	6	6.5%	*11*.*1%*	72	64.9%	39	35.1%	93	111
*Missing*	5	45.5%	*100*.*0%*	0	0.0%	*0*.*0%*	0	0.0%	*0*.*0%*	6	50.0%	6	50.0%	11	12
Gestational age (weeks)^1^															
<20	75	33.6%	*78*.*1%*	11	4.9%	*11*.*5%*	10	4.5%	*10*.*4%*	149	54.0%	127	46.0%	223	276
20 or more	68	32.2%	*73*.*1%*	20	9.5%	*21*.*5%*	5	2.4%	*5*.*4%*	116	49.6%	118	50.4%	211	234
HIV Status at ANC enrollment															
Known positive	71	36.6%	*78*.*9%*	12	6.2%	*13*.*3%*	7	3.6%	*7*.*8%*	128	55.2%	104	44.8%	194	232
New positive	72	30.0%	*72*.*7%*	19	7.9%	*19*.*2%*	8	3.3%	*8*.*1%*	137	49.3%	141	50.7%	240	278
ART initiation per clinic records															
At first ANC visit	**125**	**35.9%**	*77*.*2%*	25	7.2%	*15*.*4%*	12	3.4%	*7*.*4%*	216	53.1%	186	45.7%	348	407
After first ANC visit	**7**	**23.3%**	*70*.*0%*	2	6.7%	*20*.*0%*	1	3.3%	*10*.*0%*	20	50.0%	20	50.0%	30	40
Never received ART	**11**	**19.6%**	*64*.*7%*	4	7.1%	*23*.*5%*	2	3.6%	*11*.*8%*	25	39.7%	39	61.9%	56	63

^1^GA cut-off based on median value (19 weeks)

Engaged from care outcomes p<0.05 per chi square in bold. Facility setting (urban, semi-urban, rural) and maternal WHO stage and CD4 cell count were also examined across groups, and no differences were observed

^2^Excludes 76 cases of documented pregnancy loss, death at delivery, or maternal transfer out or relocation out of country from the original population of 510 L:TF infants

Results of crude and multivariable regression models assessing associations between maternal and facility characteristics and engagement in care among LTF women and infants, accounting for correlation between participants within health facilities, are presented in Table 5. In a multivariable model also adjusting for maternal age group, gestational age of 20+ weeks (vs. less than 20 weeks, aRR 0.82; 95% CI 0.68–0.99) and maternal ART initiation—specifically for participants who had never initiated ART (vs. ART at first ANC visit, aRR 0.54; 95% CI 0.37–0.79)—were associated with lower levels of engagement in care among the full population of LTF women. Among women with known outcomes, also adjusting for maternal knowledge of HIV status, the 25–29 year age group (aRR 0.81; 95% CI 0.67–0.97), compared with women aged 15–24 years, and gestational age of 20+ weeks (vs. less than 20 weeks, aRR 0.80; 95% CI 0.68–0.94) were associated with lower engagement, while initiating ART after the first ANC visit was associated with higher engagement (vs. ART at first ANC visit, aRR 1.12; 95% CI 1.04–1.21). Among the combined group of live infants and infants with unknown outcomes, after adjusting for age group, LTF infants of women who never received ART were less likely to have been engaged in care (vs. ART at first ANC visit, aRR 0.46; 95% CI 0.30–0.68). Limited to the group of live infants with known outcomes and adjusting for mother’s age group and knowledge of HIV status at ANC enrolment, mother not receiving ART was also associated with lower engagement in care (vs. ART at first ANC visit, aRR 0.71; 95% CI 0.54–0.91).

**Table 5:** Factors associated with engagement in care among women and infants with known outcomes and among all LTF.

	Women								Infants							
	All LTF women (n = 434)				*Women with known outcomes (n = 194)*				Live infants + infants with unknown outcomes^1^ (n = 434)				*Live infants with known outcomes (n = 189)*			
	RR	95% CI	aRR	95% CI	RR	95% CI	aRR	95% CI	RR	95% CI	aRR	95% CI	RR	95% CI	aRR	95% CI
Age group (years)																
15–24	ref				ref				ref		ref		ref			
25–29	0.91	0.77–1.07	0.91	0.77–1.09	0.84	0.67–1.07	**0.81**	**0.67–0.97**	1.10	0.89–1.37	1.08	0.87–1.34	1.06	0.89–1.26	0.97	0.81–1.18
≥30	1.18	0.67–2.06	1.17	0.64–2.13	0.89	0.61–1.30	0.88	0.58–1.33	1.26	0.96–1.64	1.24	0.99–1.56	0.89	0.73–1.09	0.84	0.67–1.05
Gestational age (weeks)																
<20	ref				ref				ref				ref			
20 or more	0.81	0.68–0.97	**0.82**	**0.68–0.99**	0.8	0.68–0.95	**0.80**	**0.68–0.94**	0.99	0.82–1.19	-	-	0.98	0.90–1.08	-	-
HIV Status at ANC enrollment																
New positive	ref				ref				ref				ref			
Known positive	0.95	0.86–1.06	-	-	0.91	0.81–1.02	0.96	0.83–1.10	1.16	0.87–1.55	-	-	1.10	0.92–1.32	1.19	0-92-1.53
Mother received ART																
First ANC visit	ref				ref				ref				ref			
After first ANC visit	1.14	0.86–1.52	1.04	0.79–1.37	1.04	0.86–1.27	**1.12**	**1.04–1.21**	0.64	0.36–1.15	0.63	0.36–1.10	0.89	0.59–1.34	0.85	0.55–1.32
Never received ART	0.64	0.47–0.88	**0.54**	**0.37–0.79**	0.89	0.67–1.17	0.79	0.56–1.11	0.50	0.35–0.73	**0.46**	**0.30–0.68**	0.83	0.63–1.09	**0.71**	**0.54–0.91**

^1^Excludes 76 cases of documented pregnancy loss, death at delivery, or maternal transfer out or relocation out of country from the original population of 510 LTF infants

## Discussion

Through a review of existing records, active patient tracing, and study interviews, we aimed to identify true levels of engagement in care and other outcomes—death, pregnancy loss, disengagement—among a population of women and infants classified as LTF from PMTCT services during the early introduction of Option B+ in Eswatini. These outcomes were able to be ascertained from existing records for less than 10% of women and infants initially LTF, while roughly half of the remaining LTF were able to be traced via phone or in the community. The study results suggest that a majority of women (62.9%) and infants (54.0%) thought to be LTF at 6-months postpartum were, in fact, engaged in care at this point in time; though, it should be noted that these results are based on outcomes ascertained among roughly half of the initial populations of LTF women (44.7%) and infants (52.0%). In addition, we found that a majority of infants reached under this study did not receive routine EID testing. Factors related to successful engagement in care among the LTF women in multivariable analyses included age—with women younger than 25 years in age somewhat more likely to be engaged—gestational age of less than 20 weeks at first ANC visit, and ART initiation, with women initiating ART after the first ANC visit experiencing higher engagement and women who never initiated ART experiencing lower engagement in care. Among LTF infants, poorer engagement was also associated with mothers not initiating ART.

This is the first published study to our knowledge that assesses outcomes among both women and infants LTF from Option B+ services. Tweya, et al. previously described current outcomes among traced women recently LTF from Option B+ care in Malawi; 40% of LTF women were successfully reached, and only 30% of these women were found to have self-transferred to another facility.[16] In another study, Kiwanuka, et al. attempted to trace 518 women who had initiated Option B+ services in Uganda approximately 2–4 years prior, 51% of whom had been classified as LTF from care. Forty six percent of the 518 women were successfully reached and interviewed; among the subset of previously LTF women that were reached, 36% were currently engaged in care in a different facility at the time of tracing.[17] Our study found a substantially higher proportion of women to be engaged in care (61%); other than differences in study settings, potential reasons for this large difference are not clear. One noteworthy difference between the Malawi research and our study is the duration between last Option B+ services and tracing—in Malawi, women were traced on an ongoing basis, within weeks after missing an appointment, while in our study all LTF patients were traced several years after their initial missed visits. In addition, the outcome in our study was engagement at 6 months postpartum—though 83 women were reached only through tracing, for whom current engagement was measured as an outcome—while the two prior studies exclusively measured current engagement, regardless of the amount of time since delivery. It is possible that 6-month engagement is in general higher than subsequent retention among Option B+ women, and in fact only 35 (42%) of the 83 women traced but not interviewed in our study reported current engagement in HIV care (Fig 1). Lastly, we note that Eswatini has a well-developed, decentralized health system and consequently the estimated ART coverage among the overall adult female population living with HIV is high (77.0%); estimated coverage among women in Malawi (69.5%) and Uganda (61.9%) is somewhat lower.[23–25]

Given that our study population is limited to a group of women and infants who were LTF from care, we advise caution in interpreting these findings in relation to results from more general Option B+ populations. Further, we note that the population of women is limited to patients newly-initiating ART in ANC; patterns of LTF and engagement in care may differ from populations that include women already receiving ART at first ANC visit. As the vast majority of previous studies of Option B+ patients have relied on LTF status as the main retention outcome measure, the entire population of patients included in our study is roughly analogous to the sub-populations not retained in most previous studies.[7] The implication of this distinction is straightforward with respect to our findings on engagement in care—for example, the high proportions of LTF women and infants found to be engaged in care in our study support an interpretation that some proportion of women and infants LTF in other previous studies were likely engaged in care as well. Less straightforward is understanding how this distinction might affect correlates of retention or engagement. Our finding of a higher risk of disengagement among women with a gestational age of 20 or more weeks at first ANC visit is consistent with the multiple studies of general Option B+ populations that found poorer retention among women initiating ART later versus earlier in pregnancy; similarly, higher engagement in care among women initiating ART after the first ANC visit in our study is consistent with prior studies finding lower retention among women initiating ART on the day of diagnosis.[7] Our finding that women in the youngest age group (15–24 years) were more likely to be engaged in care than women in an older age category (25–29 years) is, on the other hand not consistent with assessments of retention in general Option B+ populations, which have overwhelmingly found younger age associated with poorer retention. [7] The reason for this is not clear—one possible factor is higher overall rates of self-transfer among younger women, which would result in higher levels of LTF per clinic records than is measured for older women, and a likely higher level of engagement among those women LTF.

We note that the substantial proportions of LTF women (55%) and infants (48%) whose engagement outcomes were not ascertained under this study—and the fact that these women and infants are not strictly a random subset of LTF patients—prevented us from using the ascertained outcomes to provide estimates of engagement for women and infants in the overall Option B+ population, as has been done previously with general ART populations.[15,26] Further, the effectiveness of multiple imputation for estimating missing outcomes of the type examined in our study—which might be envisioned as a solution to the problem of outcomes not ascertained—is not well-established.[27]

While our findings are generally encouraging, the fact that roughly half of LTF women and infants did not have outcomes ascertained must be noted. This aspect of our results is clear limitation of the study; however, it is not inconsistent with the two previous efforts to trace women who had been receiving Option B+ services, referenced above.[16,17] Regarding the potential impact of the level of outcome ascertainment on our findings, of primary concern is the degree to which the women and infants with outcomes ascertained differed from those who did not have an outcome ascertained, and whether those differences led to a biased account of engagement in care. Comparing the populations of LTF women and infants successfully traced with the overall populations eligible for tracing, there was one characteristic that differed sharply between the eligible and successfully traced populations, respectively—the durations between HIV care enrollment and tracing. The relationship between successful tracing and duration between HIV care enrollment—at which point the participant’s telephone and home location information is collected—and the first tracing attempt should be unsurprising, as the likelihood of any given patient switching to a new phone number, and/or moving to a new home location would be expected to increase over time. Further, there is no clear likely relationship between this factor and engagement in care; therein we found no clear evidence of bias in ascertainment of outcomes among the LTF populations. It is entirely possible, however, that unmeasured factors related to engagement (e.g., disclosure of HIV status to partners) also influenced the likelihood of successful tracing and introduced bias in the assessment of outcomes.

Our findings have several implications for practice. First, while we found substantial engagement in care among live infants with outcomes ascertained, overall coverage of 6-week EID testing was low. This reflects missed opportunities for infant testing and immediate ART initiation for HIV-infected infants, as well as the multiple barriers to conducting routine EID testing.[28] Given the critical need to provide ART as early as possible to HIV-infected infants, strengthening the provision of routine EID testing is an urgent priority.[29] Second, concerns regarding disproportionately high loss to follow-up in Option B+ PMTCT services, as documented in previous research, should be informed by these findings, which point to a high degree of self-transferring or undocumented return to care rather than disengagement from PMTCT or ART.[7] Mobility within the context of PMTCT among pregnant and postpartum women has been discussed in previous literature, though the degree to which this factor contributes to LTF among Option B+ populations is not well-established.[18,30–34] In Eswatini—which is known to have a highly mobile population—mobility of patients and limitations to existing data systems present a widespread challenge to understanding true levels of engagement in care, and factors related to risk of disengagement.[31,32] Particularly high mobility during pregnancy and after delivery could also have adversely impacted our ability to ascertain outcomes via tracing, and may in part underlie the similar rates of tracing success in other studies of Option B+ populations, lower in relation to many tracing studies of general ART populations.[16,17,35] In addition, findings of our exploratory analyses provide preliminary results for practice and future investigation. Seemingly counterintuitive is our finding that engagement in care was somewhat higher among LTF women with pregnancy loss or infant death at delivery. However, noting that the women in the study population were all LTF from their ANC facility, this suggests that these women were more likely than other LTF women to return to care or relocate their HIV care to another facility. Therefore, improved facility transfer services and care coordination may be needed for women following a pregnancy loss or early infant death. These analyses also pointed towards somewhat better retention outcomes for infants—engagement in care, 6-week EID testing—of women who were engaged in care and, conversely, a relationship between maternal and infant disengagement from services. As these mother-baby pairs entirely disengaged from PMTCT services are at a critically elevated risk of MTCT and other adverse outcomes, providers and health systems should prioritize the identification of these women and infants and rapidly engage them back into care. A final clear implication of our findings is that efforts are needed to strengthen data systems to better track and understand outcomes across the PMTCT care cascade. The critical need to improve systems to allow for tracking of patients across time and service points—for example through implementation of a national unique identifier system—is widely understood, and numerous countries including Eswatini are in the process of implementing such a system.[1,32,36,37] However, there were particular gaps in national tools that resulted in missed opportunities to document outcomes, which could be addressed without need for linked national data incorporating unique identifiers. For example, the absence of structured documentation of pregnancy outcomes for ANC patients resulted in substantial undocumented, unreported pregnancy loss. We note that nearly 10% of all LTF infant cases—and approximately 20% of the LTF infant cases with an outcome ascertained—were undocumented pregnancy losses, so this gap poses an important challenge to measuring retention of infants across the PMTCT care cascade, and it cannot be remedied through a system of linking patients across services using unique identifiers. The system for documenting transfers of PMTCT care across health facilities in Eswatini, consistent with global guidance, is incomplete and inconsistent.[38]

There are limitations to this study that should be noted. First and foremost, as highlighted above, under this study we were unable to ascertain outcomes for approximately half of the women and infants LTF from Option B+ care. We have some indication of how these patients might differ from those with outcomes ascertained under this study—including potentially important factors such as maternal ART initiation, and factors that seems to present less of a threat of selection bias, in particular the time between HIV care enrollment and study tracing. Another noteworthy limitation is the self-reported nature of engagement in care data for a subset of women and infants (via maternal report), i.e., those reached only via tracing or interviewed but not providing patient-held records for abstraction. Further, specifically for the women reached by tracing but not interviewed, this information provides only an indication of self-reported *current* and *most recent* engagement in care; it does not directly address engagement at 6-months after delivery. This may have resulted in a somewhat inaccurate level of engagement in care for women and infants. In particular, recognizing that the study population was made up of women with reproductive potential, it is likely that many women again became pregnant, subsequent to the pregnancy coinciding with Option B+ implementation, and in some cases this may have prompted women who had disengaged to return to care. Lastly, generalizability of our findings may be limited by the setting—Eswatini, which has particularly high HIV prevalence as well as high ART and PMTCT coverage—as well as fact that the study population was among the first in Eswatini to receive Option B+, and therefore aspects of services were not longstanding and perhaps not well-established.

## Conclusions

Overall, our findings among women and infants with outcomes ascertained point to higher engagement in care among women and infants under Option B+ than is captured in assessments based solely on facility-based records. However, important programmatic gaps remain, including HIV testing among exposed infants. This study also highlights shortcomings in current systems for documenting HIV services and patient outcomes, raises concerns about adequate support for women with pregnancy loss or death of an infant, and helps us point to the subgroup of mother-infant pairs disengaged from PMTCT services and at high risk of adverse outcomes. Measures to strengthen routine data systems across PMTCT services—including services provided across multiple facilities—and better ensure follow-up of women and HEI are critically needed to effectively monitor, and improve, outcomes of women living with HIV and their infants.

## Supporting information

S1 Table Characteristics of 10 study health facilities in Eswatini at time of study initiation. (XLSX)

S2 Table Engagement among women by pregnancy loss and infant death, limited to women with known outcomes in SG+ (n = 194). (XLSX)

S3 Table Engagement among women by pregnancy loss and infant death, including all LTF women (n = 434). (XLSX)

S4 Table Infant outcomes by mother outcomes and ascertainment of mother outcome. (XLSX)

S5 Table Engagement among infants by mother engagement in care outcome, including all live infants with known outcomes (n = 189). (XLSX)

S6 Table Engagement among infants by mother LTF outcome, including all live infants with known outcomes (n = 189). (XLSX)

S7 Table Six week infant testing by mother LTF and/or engagement in care outcome among LTF infants whose mother completed an SG+ study survey and provided relevant information (n = 87). (XLSX)

S1 Appendix Tracing interview (English). (DOCX)

S2 Appendix Tracing interview (SiSwati). (DOCX)

S3 Appendix Study interview (English). (DOCX)

S4 Appendix Study interview (SiSwati). (DOCX)

S1 File Codebook for analysis. (XLSX)

S2 File Data used for this paper. (CSV)
