# Supplementary material for: Engagement in care among women and their infants lost to follow-up under Option B+ in eSwatini
Source: PLoS One. 2019 Oct 30;14(10):e0222959. doi: 10.1371/journal.pone.0222959 (PMC6821080; doi:10.1371/journal.pone.0222959)
Supplement: S1 Appendix — (DOCX) [file pone.0222959.s008.docx]

| **STANDARD TRACING FORM**  **COMPLETE ALL OF THE INFORMATION ON THIS PAGE BEFORE ATTEMPTING TO CONTACT THE PATIENT.** | | |
| --- | --- | --- |
| **A01** | Facility Name | _________________________________________ |
| **A02** | Maternal or infant LTF | □Maternal LTF  □Infant LTF  □Maternal and infant LTF |
| **A03** | Maternal Pre-ART/ART Number: | _________________________________________ |
| **A04** | Mother’s Name | _________________________________________ |
| **A05** | Date of mother’s first HIV care visit: | \|  \|  \|  \|  \|  \|  \|  \|  \|  \|  \| \| --- \| --- \| --- \| --- \| --- \| --- \| --- \| --- \| --- \| --- \| \| **Day** \| \|  \| **Month** \| \|  \| **Year** \| \| \| \| \| |
| **A06** | Date of maternal ART initiation at clinic:  □ Check if never started ART | \|  \|  \|  \|  \|  \|  \|  \|  \|  \|  \| \| --- \| --- \| --- \| --- \| --- \| --- \| --- \| --- \| --- \| --- \| \| **Day** \| \|  \| **Month** \| \|  \| **Year** \| \| \| \| \| |
| **A07** | Date mother last seen at facility: | \|  \|  \|  \|  \|  \|  \|  \|  \|  \|  \| \| --- \| --- \| --- \| --- \| --- \| --- \| --- \| --- \| --- \| --- \| \| **Day** \| \|  \| **Month** \| \|  \| **Year** \| \| \| \| \| |
| **A08** | Estimated delivery date: | \|  \|  \|  \|  \|  \|  \|  \|  \|  \|  \| \| --- \| --- \| --- \| --- \| --- \| --- \| --- \| --- \| --- \| --- \| \| **Day** \| \|  \| **Month** \| \|  \| **Year** \| \| \| \| \| |
| **A09** | Infant’s CWC Number: | _________________________________________ |
| **A10** | Date infant last seen at facility: | \|  \|  \|  \|  \|  \|  \|  \|  \|  \|  \| \| --- \| --- \| --- \| --- \| --- \| --- \| --- \| --- \| --- \| --- \| \| **Day** \| \|  \| **Month** \| \|  \| **Year** \| \| \| \| \|   □Never seen |
| **A11** | Last infant visit type | □ 7-10 day visit  □ 6-week  □ 10-week  □ 14-week  □ 6-month  □ 9-month  □ 12-month  □ HIV clinic visit  □ Not applicable |

| **NO** | **QUESTION** | **ATTEMPT 1** | **ATTEMPT 2** | **ATTEMPT 3** |
| --- | --- | --- | --- | --- |
| **B01** | Staff initials | __________________ | __________________ | __________________ |
| **B02** | Phone call or community visit: | □ Phone call  □Community visit | □ Phone call  □Community visit | □ Phone call  □Community visit |
| **B03** | Date of contact attempt: | __ __ / __ __ / __ __ | __ __ / __ __ / __ __ | __ __ / __ __ / __ __ |
| **B04** | Attempting to contact: | □ Patient  □ Informant | □ Patient  □ Informant | □ Patient  □ Informant |
| **B05** | Outcome code: | ___________________ | ___________________ | ___________________ |

| **NO** | | **QUESTION** | **ATTEMPT 4** | **ATTEMPT 5** | **ATTEMPT 6** |
| --- | --- | --- | --- | --- | --- |
| **B01** | | Staff initials | __________________ | __________________ | __________________ |
| **B02** | | Phone call or community visit: | □ Phone call  □Community visit | □ Phone call  □Community visit | □ Phone call  □Community visit |
| **B03** | | Date of contact attempt: | __ __ / __ __ / __ __ | __ __ / __ __ / __ __ | __ __ / __ __ / __ __ |
| **B04** | Attempting to contact: | | □ Patient  □ Informant | □ Patient  □ Informant | □ Patient  □ Informant |
| **B05** | | Outcome code: | ___________________ | ___________________ | ___________________ |

| **NO** | **QUESTION** | **ATTEMPT 7** | **ATTEMPT 8** | **ATTEMPT 9** |
| --- | --- | --- | --- | --- |
| **B01** | Staff initials | __________________ | __________________ | __________________ |
| **B02** | Phone call or community visit: | □ Phone call  □Community visit | □ Phone call  □Community visit | □ Phone call  □Community visit |
| **B03** | Date of contact attempt: | __ __ / __ __ / __ __ | __ __ / __ __ / __ __ | __ __ / __ __ / __ __ |
| **B04** | Attempting to contact: | □ Patient  □ Informant | □ Patient  □ Informant | □ Patient  □ Informant |
| **B05** | Outcome code: | ___________________ | ___________________ | ___________________ |

**Community tracing outcome codes:**

**CM-PAT:**  Successfully reached patient [Script read]

**CM-INF:** Successfully reached informant  [Script read]

**CM-CB:** Told to come back  [fill in come back time]

**CM-REF:** Refused to converse  {TERMINAL}

**CM-WRAD:** Wrong address for participant

**CM-NOA:**  No answer; reach home with no answer

**Phone tracing outcome codes:**

**PH-PAT:**  Successfully reached patient [Script read]

**PH-INF:** Successfully reached informant  [Script read]

**PH-CB:** Told to call back  [fill in Call back time]

**PH-NEW#:** Referred to new number  [fill in referral number]

**PH-REF:** Refused to converse {TERMINAL}

**PH-WR#:** Wrong number for participant

**PH-NOA:** No answer; phone rings without an answer

**PH-BZ#:** Busy number

**PH-INV:** Invalid number; not a working number

| **MATERNAL TRACING**  **(*Skip this section of mother is not LTF)*** | | |
| --- | --- | --- |
| **C01** | Since [DATE IN A07] have you received HIV care? | □Yes  □No **(GO TO C04)**  □Refused to answer **(GO TO C04)** |
| **C02** | What is the date that you most recently received HIV care? | **C02.1** ***Prompt if not known:***  __ __ - __ __ __ __ **(GO TO C03)** □*More than three months ago?*  MM YYYY □ *1-3 months ago?*  □ *Less than 1 month ago?*  □Refused to answer **(GO TO C03)** □*Less than one week ago?*  □Unknown □*Less than one day ago?*  □*Refused to answer*  □*Unknown* |
| **C03** | What is the name of the clinic where you most recently received HIV care? | Name of Clinic: ___________________  □Refused to answer  □Unknown |
| **C04** | Have you ever taken ART? | □Yes  □No **(GO TO D01)**  □Refused to answer **(GO TO D01)** |
| **C05** | Since [DATE IN A07] have you received ART? | □Yes  □No **(GO TO D01)**  □Refused to answer **(GO TO D01)** |
| **C06** | What is the date that you most recently took ART? | **C06.1** ***Prompt if not known:***  __ __ - __ __ __ __ **(GO TO D01)** □*More than three months ago?*  DD YYYY □ *1-3 months ago?*  □ *Less than 1 month ago?*  □Refused to answer **(GO TO D01)** □*Less than one week ago?*  □Unknown □*Less than one day ago?*  □*Refused to answer*  □*Unknown* |

| **INFANT TRACING**  **(*Skip this section of infant is not LTF)*** | | |
| --- | --- | --- |
| **D01** | Did you deliver a baby during the pregnancy for which you attended HIV care at [CLINIC in A01] around [DATE IN A06]? | □Yes **(GO TO D03)**  □No  □Refused to answer **(GO TO D03)** |
| **D02** | Why was the baby not delivered? | □Infant died during pregnancy **(END)**  □Infant died during delivery **(END)**  □Other (Specify:___________________________________)  □Refused to answer  □Unknown |
| **D03** | What is the baby’s DOB? | __ __ / __ __ / __ __  □ N/A  □Refused to answer  □Unknown |
| **D04** | Infant health status: | □Yes, infant alive and healthy  □No, infant alive but sick  □No, infant died <6 weeks after delivery  □Infant died >6 weeks after delivery **(END)**  □Unknown  □Refused to answer  □Other: ____________ |
| **D05** | Has your infant ever attended an ‘Under-Five’ clinic? | □Yes  □No **(END)**  □Refused to answer **(END)**  □Unknown |
| **D06** | How old was your infant the last time s/he attended the ‘Under-Five’ clinic?  *Only record weeks if infant was under 2 months old.* | ***_______*** years □Refused to answer  **_______** months □Unknown  **________** weeks |
| **D07** | At which ‘Under-Five’ clinic did your infant most recently receive care? | Name of Clinic: ___________________  □Refused to answer  □Unknown |

| **INFORMANT** | | |
| --- | --- | --- |
| **E01** | Informant’s relationship to woman: | ___________________ |
| **E02** | When did you last see the woman? | **E02.1** ***Prompt if not known:***  __ __ - __ __ __ __ **(GO TO E03)** □*More than12 mos. ago?*  MM YYYY □*In last 12 mos.?*  □*In last 6 mos.?*  □*In last 3 mos.?*  □Refused to answer **(GO TO E03)** □*In last 1 mos.?*  □Unknown □*Refused to answer*  □*Unknown* |
| **E03** | Is the woman still in Swaziland as far as you know? | □Yes  □No **(E05)**  □Unknown  □Refused to answer |
| **E04** | What area of Swaziland is she located in as far as you know? | ____________________________  □Unknown  □Refused to answer |
| **E05** | As far as you are aware, is she alive and healthy?  [Probe for answer category as needed]? | □Yes, patient alive and healthy **(E08)**  □No, patient alive but sick **(E08)**  □No, patient died  □Unknown **(E08)**  □Refused to answer **(E08)**  □Other: ____________ **(E08)** |
| **E06** | What date did the woman pass away? | **E06.1** ***Prompt if not known:***  __ __ - __ __ __ __ **(GO TO E07)** □*More than12 mos. ago?*  MM YYYY □*In last 12 mos.?*  □*In last 6 mos.?*  □*In last 3 mos.?*  □Refused to answer **(GO TO E07)** □*In last 1 mos.?*  □Unknown □*Refused to answer*  □*Unknown* |
| **E07** | What was the cause of the woman’s death? | □Disease or illness  □Injury, accident, or trauma  □Relating to pregnancy or childbirth  □Suicide  □Other __________________________  □Unknown  □Refused to answer |
| **E08** | Did the woman have a baby around [DATE IN A08]? | □Yes  □No **(END)**  □Unknown **(END)**  □Refused to answer **(END)** |
| **E09** | As far as you are aware, is the baby alive and healthy?  [Probe for answer category as needed] | □Yes, infant alive and healthy  □No, infant alive but sick  □No, infant died during pregnancy  □No, infant died during delivery  □No, infant died after delivery  □Unknown  □Refused to answer  □Other: _________________ |
