# Supplementary material for: Engagement in care among women and their infants lost to follow-up under Option B+ in eSwatini
Source: PLoS One. 2019 Oct 30;14(10):e0222959. doi: 10.1371/journal.pone.0222959 (PMC6821080; doi:10.1371/journal.pone.0222959)
Supplement: S2 Appendix — (DOCX) [file pone.0222959.s009.docx]

| **STANDARD TRACING FORM**  **COMPLETE ALL OF THE INFORMATION ON THIS PAGE BEFORE ATTEMPTING TO CONTACT THE PATIENT.** | | |
| --- | --- | --- |
| **A01** | Facility Name | _________________________________________ |
| **A02** | Maternal or infant LTF | □Maternal LTF  □Infant LTF  □Maternal and infant LTF |
| **A03** | Maternal Pre-ART/ART Number: | _________________________________________ |
| **A04** | Mother’s Name | _________________________________________ |
| **A05** | Date of mother’s first HIV care visit: | \|  \|  \|  \|  \|  \|  \|  \|  \|  \|  \| \| --- \| --- \| --- \| --- \| --- \| --- \| --- \| --- \| --- \| --- \| \| **Day** \| \|  \| **Month** \| \|  \| **Year** \| \| \| \| \| |
| **A06** | Date of maternal ART initiation at clinic:  □Check if never started ART | \|  \|  \|  \|  \|  \|  \|  \|  \|  \|  \| \| --- \| --- \| --- \| --- \| --- \| --- \| --- \| --- \| --- \| --- \| \| **Day** \| \|  \| **Month** \| \|  \| **Year** \| \| \| \| \| |
| **A07** | Date mother last seen at facility: | \|  \|  \|  \|  \|  \|  \|  \|  \|  \|  \| \| --- \| --- \| --- \| --- \| --- \| --- \| --- \| --- \| --- \| --- \| \| **Day** \| \|  \| **Month** \| \|  \| **Year** \| \| \| \| \| |
| **A08** | Estimated delivery date: | \|  \|  \|  \|  \|  \|  \|  \|  \|  \|  \| \| --- \| --- \| --- \| --- \| --- \| --- \| --- \| --- \| --- \| --- \| \| **Day** \| \|  \| **Month** \| \|  \| **Year** \| \| \| \| \| |
| **A09** | Infant’s CWC Number: | _________________________________________ |
| **A10** | Date infant last seen at facility: | \|  \|  \|  \|  \|  \|  \|  \|  \|  \|  \| \| --- \| --- \| --- \| --- \| --- \| --- \| --- \| --- \| --- \| --- \| \| **Day** \| \|  \| **Month** \| \|  \| **Year** \| \| \| \| \|   □Never seen |
| **A11** | Last infant visit type | □7-10 day visit  □6-week  □10-week  □14-week  □6-month  □9-month  □12-month  □HIV clinic visit  □Not applicable |

| **NO** | **QUESTION** | **ATTEMPT 1** | **ATTEMPT 2** | **ATTEMPT 3** |
| --- | --- | --- | --- | --- |
| **B01** | Staff initials | __________________ | __________________ | __________________ |
| **B02** | Phone call or community visit: | □Phone call  □Community visit | □Phone call  □Community visit | □Phone call  □Community visit |
| **B03** | Date of contact attempt: | __ __ / __ __ / __ __ | __ __ / __ __ / __ __ | __ __ / __ __ / __ __ |
| **B04** | Attempting to contact: | □Patient  □Informant | □Patient  □Informant | □Patient  □Informant |
| **B05** | Outcome code: | ___________________ | ___________________ | ___________________ |

| **NO** | | **QUESTION** | **ATTEMPT 4** | **ATTEMPT 5** | **ATTEMPT 6** |
| --- | --- | --- | --- | --- | --- |
| **B01** | | Staff initials | __________________ | __________________ | __________________ |
| **B02** | | Phone call or community visit: | □Phone call  □Community visit | □Phone call  □Community visit | □Phone call  □Community visit |
| **B03** | | Date of contact attempt: | __ __ / __ __ / __ __ | __ __ / __ __ / __ __ | __ __ / __ __ / __ __ |
| **B04** | Attempting to contact: | | □Patient  □Informant | □Patient  □Informant | □Patient  □Informant |
| **B05** | | Outcome code: | ___________________ | ___________________ | ___________________ |

| **NO** | **QUESTION** | **ATTEMPT 7** | **ATTEMPT 8** | **ATTEMPT 9** |
| --- | --- | --- | --- | --- |
| **B01** | Staff initials | __________________ | __________________ | __________________ |
| **B02** | Phone call or community visit: | □Phone call  □Community visit | □Phone call  □Community visit | □Phone call  □Community visit |
| **B03** | Date of contact attempt: | __ __ / __ __ / __ __ | __ __ / __ __ / __ __ | __ __ / __ __ / __ __ |
| **B04** | Attempting to contact: | □Patient  □Informant | □Patient  □Informant | □Patient  □Informant |
| **B05** | Outcome code: | ___________________ | ___________________ | ___________________ |

**Community tracing outcome codes:**

**CM-PAT:** Successfully reached patient [Script read]

**CM-INF:** Successfully reached informant  [Script read]

**CM-CB:** Told to come back  [fill in come back time]

**CM-REF:** Refused to converse  {TERMINAL}

**CM-WRAD:** Wrong address for participant

**CM-NOA:** No answer; reach home with no answer

**Phone tracing outcome codes:**

**PH-PAT:**  Successfully reached patient [Script read]

**PH-INF:** Successfully reached informant  [Script read]

**PH-CB:** Told to call back  [fill in Call back time]

**PH-NEW#:** Referred to new number  [fill in referral number]

**PH-REF:** Refused to converse {TERMINAL}

**PH-WR#:** Wrong number for participant

**PH-NOA:** No answer; phone rings without an answer

**PH-BZ#:** Busy number

**PH-INV:** Invalid number; not a working number

| **MATERNAL TRACING**  **(*Skip this section of mother is not LTF)*** | | |
| --- | --- | --- |
| **C01** | Kusukela ku [Lusuku loluse A07]? | □Yes  □No **(GO TO C04)**  □Refused to answer **(GO TO C04)** |
| **C02** | Nguluphi lusuku lapho Ugcine khona kutfola lusito nge HIV? | **C02.1*Prompt if not known:***  __ __ - __ __ __ __ **(GO TO C03)** □*More than three months ago?*  MM YYYY □ *1-3 months ago?*  □ *Less than 1 month ago?*  □Refused to answer **(GO TO C03)** □*Less than one week ago?*  □Unknown □*Less than one day ago?*  □*Refused to answer*  □*Unknown* |
| **C03** | Litsini libito lekliniki lapho ugcine khona kutfola lusito nge HIV? | Name of Clinic: ___________________  □Refused to answer  □Unknown |
| **C04** | Wake wawatsatsa yini ema ARVs? | □Yes  □No **(GO TO D01)**  □Refused to answer **(GO TO D01)** |
| **C05** | Kusukela nga [Lusuku ku A07] uke wawatfola yini ema ARV? | □Yes  □No **(GO TO D01)**  □Refused to answer **(GO TO D01)** |
| **C06** | Nguluphi lusuku locinisekile kutsi ngulo logcine ngalo kutsatsa ema ARVs? | **C06.1*Prompt if not known:***  __ __ - __ __ __ __ **(GO TO D01)** □*More than three months ago?*  DD YYYY □ *1-3 months ago?*  □ *Less than 1 month ago?*  □Refused to answer**(GO TO D01)** □*Less than one week ago?*  □Unknown □*Less than one day ago?*  □*Refused to answer*  □*Unknown* |

| **INFANT TRACING**  **(*Skip this section of infant is not LTF)*** | | |
| --- | --- | --- |
| **D01** | Waphumelela yini kubeleka kulokutetfwala nawuya e [Kliniki A01] ngakubo mhlaka [lusuku in A06]? | □Yes**(GO TO D03)**  □No  □Refused to answer **(GO TO D03)** |
| **D02** | Kwentiwa yini wangabelekwa umntfwana? | □Infant died during pregnancy **(END)**  □Infant died during delivery **(END)**  □Other (Specify:___________________________________)  □Refused to answer  □Unknown |
| **D03** | Lutsini lusuku lwekutalwa kwemntfwana? | __ __ / __ __ / __ __  □N/A  □Refused to answer  □Unknown |
| **D04** | Simo semphilo seluswane: | □Yes, infant alive and healthy  □No, infant alive but sick  □No, infant died <6 weeks after delivery  □Infant died >6 weeks after delivery **(END)**  □Unknown  □Refused to answer  □Other: ____________ |
| **D05** | Uke waya yini umntfwana wakho “ekliniki yebantfwana labangaphansi kwa 5”? | □Yes  □No **(END)**  □Refused to answer **(END)**  □Unknown |
| **D06** | Abenganani umntfwana wakho nakagcina kuya ekliniki yebantfwana labangaphansi kwa 5”?  *Only record weeks if infant was under 2 months old.* | ***_______*** years □Refused to answer  **_______** months □Unknown  **________** weeks |
| **D07** | Uye kuyiphi ikliniki yebantfwana labangaphansi kwa 5” lapho agcine khona? | Name of Clinic: ___________________  □Refused to answer  □Unknown |
| **INFORMANT** | | |
| **E01** | Buhlobo balobutwako nemfati: | ___________________ |
| **E02** | Ugcine nini kumbona lomfati? | **E02.1*Prompt if not known:***  __ __ - __ __ __ __ **(GO TO E03)** □*More than12 mos. ago?*  MM YYYY □*In last 12 mos.?*  □*In last 6 mos.?*  □*In last 3 mos.?*  □Refused to answer **(GO TO E03)** □*In last 1 mos.?*  □Unknown □*Refused to answer*  □*Unknown* |
| **E03** | Lomfati usase Swatini yini ngekwati kwakho? | □Yes  □No **(E05)**  □Unknown  □Refused to answer |
| **E04** | Ungakuphi lapha eSwatini ngekwati kwakho? | ____________________________  □Unknown  □Refused to answer |
| **E05** | Ngekwati kwakho, uyaphila yini usekhona? | □Yes, patient alive and healthy **(E08)**  □No, patient alive but sick**(E08)**  □No, patient died  □Unknown **(E08)**  □Refused to answer **(E08)**  □Other: ____________ **(E08)** |
| **E06** | Wendlule ngaluphi lusuku emhlabeni lomfati? | **E06.1*Prompt if not known:***  __ __ - __ __ __ __ **(GO TO E07)** □*More than12 mos. ago?*  MM YYYY □*In last 12 mos.?*  □*In last 6 mos.?*  □*In last 3 mos.?*  □Refused to answer **(GO TO E07)** □*In last 1 mos.?*  □Unknown □*Refused to answer*  □*Unknown* |
| **E07** | Kwabayini leyabanga kutsi ashone lomfati? | □Disease or illness  □Injury, accident, or trauma  □Relating to pregnancy or childbirth  □Suicide  □Other __________________________  □Unknown  □Refused to answer |
| **E08** | Abenemntfwana yini ngabo [Lusuku A08] lomfati? | □Yes  □No **(END)**  □Unknown**(END)**  □Refused to answer **(END)** |
| **E09** | Ngekwati kwakho ukhona yini lomntfwana, uyaphila?  [Probe for answer category as needed] | □Yes, infant alive and healthy  □No, infant alive but sick  □No, infant died during pregnancy  □No, infant died during delivery  □No, infant died after delivery  □Unknown  □Refused to answer  □Other: _________________ |
