# Supplementary material for: Engagement in care among women and their infants lost to follow-up under Option B+ in eSwatini
Source: PLoS One. 2019 Oct 30;14(10):e0222959. doi: 10.1371/journal.pone.0222959 (PMC6821080; doi:10.1371/journal.pone.0222959)
Supplement: S3 Appendix — (DOCX) [file pone.0222959.s010.docx]

| **TRACING QUESTIONNAIRE**  **COMPLETE ALL OF THE INFORMATION ON THIS PAGE BEFORE BEGINNING THE INTERVIEW.** | | | |
| --- | --- | --- | --- |
|  | PMTCT enrolment clinic | Siteki PHU | 1 |
|  |  | MSF Matsapha | 2 |
|  |  | Lamvelase Clinic | 3 |
|  |  | Siphofaneni Clinic | 4 |
|  |  | RFM Hospital | 5 |
|  |  | King Sobhuza II PHU | 6 |
|  |  | Mankayane PHU | 7 |
|  |  | Luyengo PHU | 8 |
|  |  | FLAS Manzini | 9 |
|  |  | Mbikwakhe Clinic | 10 |
|  | Date of first HIV care visit: | \|  \|  \|  \|  \|  \|  \|  \|  \|  \|  \| \| --- \| --- \| --- \| --- \| --- \| --- \| --- \| --- \| --- \| --- \| \| **Day** \| \|  \| **Month** \| \|  \| **Year** \| \| \| \| \| | |
|  | Maternal or infant LTF at clinic [T01] | Maternal LTF | 1***🡪 T06*** |
|  |  | Infant LTF | 2 |
|  |  | Maternal and infant LTF | 3 |
|  | Infant delivery date  *From clinic records or Standard Tracing Form [D01 and D03], where available* | \|  \|  \| \|  \| \|  \|  \| \|  \| \|  \|  \|  \|  \| \| --- \| --- \| --- \| --- \| --- \| --- \| --- \| --- \| --- \| --- \| --- \| --- \| --- \| --- \| \| **Day** \| \|  \| \| **Month** \| \| \|  \| \| **Year** \| \| \| \| \| | |
|  |  | No delivery | *99* |
|  | Infant health status  *From clinic records or Standard Tracing Form [D02], where available* | Infant alive and healthy | 1 |
|  |  | Infant sick | 2 |
|  |  | Infant died during pregnancy | 3 |
|  |  | Infant died during delivery | 4 |
|  |  | Infant died <6 weeks after delivery | 5 |
|  |  | Infant died >6 weeks after delivery | 6 |
|  |  | Other: _________________ | *7* |
|  |  | *Don’t know* | *88* |
|  |  | *Refusal* | *99* |

|  | Interviewer code | \|  \|  \|  \| \| --- \| --- \| --- \| | | |
| --- | --- | --- | --- | --- | --- | --- | --- |
|  | SID NUMBER | \|  \|  \| **---** \|  \|  \|  \| \| --- \| --- \| --- \| --- \| --- \| --- \| | | |
| **T07** | Date of interview | \|  \|  \|  \|  \|  \|  \|  \|  \|  \|  \| \| --- \| --- \| --- \| --- \| --- \| --- \| --- \| --- \| --- \| --- \| \| **Day** \| \|  \| **Month** \| \|  \| **Year** \| \| \| \| \| | | |
|  | ***Prompt:*** Patient consented to participate? | Yes | 1 | ***If 2 🡪 END*** |
|  |  | No | 2 |  |
| **T08** | Interview start time: | \|  \|  \| **:** \|  \|  \| \| --- \| --- \| --- \| --- \| --- \| \| **Day** \| \|  \| **Month** \| \| | | |

**INTERVIEWER:** Thank you for agreeing to participate in our study. You can choose not to answer any questions that you do not want to. You may also ask me to explain questions if you do not understand them. You may also stop the interview at any time if you decide you no longer want to participate. Please remember that your responses to these questions are identified only by number and will be kept confidential. Your name does not appear on this questionnaire.

| **SeCTION A. DEMOGRAPHICS** | | | | |
| --- | --- | --- | --- | --- |
| **No.** | **QUESTIONS & INSTRUCTIONS** | **RESPONSES** | | **skips** |
| **INTERVIEWER:** Let us start with some questions about you and your household. | | | | |
|  | How much schooling have you completed?  *Do not read options aloud. Circle one response.* | None | 1 |  |
|  |  | Some primary school | 2 |  |
|  |  | Completed primary school | 3 |  |
|  |  | Some secondary school | 4 |  |
|  |  | Completed secondary school | 5 |  |
|  |  | Some high school | 6 |  |
|  |  | Completed high school | 7 |  |
|  |  | Some tertiary education | 8 |  |
|  |  | Completed tertiary education | 9 |  |
|  |  | *Don’t know* | *88* |  |
|  |  | *Refusal* | *99* |  |
|  | What is the nature of your current employment?  *Do not read options aloud. Circle all that apply.* | Self-employed | 1 |  |
|  |  | Employed full-time | 2 |  |
|  |  | Employed part-time | 3 |  |
|  |  | Not currently working | 4 |  |
|  |  | *Don’t know* | *88* |  |
|  |  | *Refusal* | *99* |  |
|  | What is the PRIMARY source of income for your household?  *Circle one response.* | Self-employment | 1 |  |
|  |  | Full-time employment | 2 |  |
|  |  | Part-time employment | 3 |  |
|  |  | Informal employment | 4 |  |
|  |  | Social grant | 5 |  |
|  |  | Pension | 6 |  |
|  |  | Income from other household members | 7 |  |
|  |  | Other type of grant, specify: ______________________________ | 8 |  |
|  |  | Other, specify: ______________________________ | 9 |  |
|  |  | None | 10 |  |
|  |  | *Don’t know* | *88* |  |
|  |  | *Refusal* | *99* |  |
|  | Do you or any members of your household own any of the following:    *Read the list, circle item if owned. Multiple responses are possible.* | A bicycle | 1 |  |
|  |  | A motorbike/motor scooter | 2 |  |
|  |  | A car | 3 |  |
|  |  | A watch | 4 |  |
|  |  | A radio | 5 |  |
|  |  | A television | 6 |  |
|  |  | A refrigerator | 7 |  |
|  |  | Agricultural land | 8 |  |
|  |  | A house | 9 |  |
|  |  | A mobile phone | 10 |  |
|  |  | A bed | 11 |  |
|  |  | *Don’t know* | *88* |  |
|  |  | *Refusal* | *99* |  |
|  | How long have you lived in your current household?  *Enter number of years in the space provided. If woman responds a value less than one year (e.g., 6 months), enter 01. If woman answers “Don’t know”, enter 88. If woman refuses to answer, enter 99.* | \|  \|  \| \| --- \| --- \| \| Years \| \| | |  |
|  | How many adults and how many children live in your household? | \|  \|  \| \| --- \| --- \| \| Adults \| \| | |  |
|  |  | \|  \|  \| \| --- \| --- \| \| Children \| \| | |  |
|  | In the last 12 months, how many times have you been away from your home for more than one month at a time?  *Enter number of times in the space provided. If woman answers “None”, enter 00 “Don’t know”, enter 88. If woman refuses to answer, enter 99.* | \|  \|  \| \| --- \| --- \| \| Times \| \| | |  |
|  | “Have you ever been divorced, or are you a widow?”  *Multiple responses are possible.* | Yes, divorced | 1 |  |
|  |  | Yes, widow | 2 |  |
|  |  | No | 3 |  |
|  |  | *Refusal* | *99* |  |
|  | Are you currently in a relationship? | Yes | 1 | ***2 🡪 A13*** |
|  |  | No | 2 |  |
|  |  | *Refusal* | *99* |  |
|  | Are you currently married? | Yes | 1 |  |
|  |  | No | 2 |  |
|  |  | *Refusal* | *99* |  |
|  | Are you currently living with your partner? | Yes | 1 |  |
|  |  | No | 2 |  |
|  |  | *Refusal* | *99* |  |
|  | How long have you been in a relationship with your current partner?  *Enter number of months and years in the spaces provided. If woman responds a value less than one month (e.g., 3 days, or 2 weeks), enter 01 Month and 00 Years. If woman answers “Don’t know”, enter 88 months and 88 years. If woman refuses to answer, enter 99 months and 99 years.* | \|  \|  \| \| --- \| --- \| \| Months \| \| \|  \|  \| \| Years \| \| | |  |
|  | Have you disclosed you HIV status toanyone? | Yes | 1 | ***2🡪 Section B*** |
|  |  | No | 2 |  |
|  |  | *Refusal* | *99* |  |

|  | To whom have you disclosed your HIV status.  *Select all that apply* | Partner | 1 |  |
| --- | --- | --- | --- | --- |
|  |  | Another sexual partner | 2 |  |
|  |  | Mother or father | 3 |  |
|  |  | Sibling | 4 |  |
|  |  | Other family member | 5 |  |
|  |  | Friend | 6 |  |
|  |  | Have made status known publicly | 7 |  |
|  |  | Other (specify: _______________) | 2 |  |
|  |  | *Refusal* | *99* |  |

| **SeCTION B. MATERNAL CARE**  ***(For all women)*** | | | | | | | | | |
| --- | --- | --- | --- | --- | --- | --- | --- | --- | --- |
| **No.** | **QUESTIONS & INSTRUCTIONS** | | **RESPONSES** | | | | | | **skips** |
| **INTERVIEWER:** Now I would like to ask you some questions about your health care. Our records indicate that you were pregnant around [DATE FROM T02]. | | | | | | | | | |
| **INTERVIEWER:** From the pregnancy around [DATE FROM T02],did you deliver a live baby?  *Refer to T04.* | | | Yes | | | | 1 | | ***If 2 🡪 B02*** |
|  |  |  | No | | | | 2 | |  |
|  | Did you deliver this pregnancy at home or in a facility? | | Delivered at home  *Specify home area:*  *______________________________* | | | | 1 | |  |
|  |  |  | Delivered at facility  *Specify delivery facility:*  *______________________________* | | | | 2 | |  |
|  |  |  | *Refusal* | | | | *99* | |  |
|  | Have you been pregnant since [PREGNANCY FROM DATE IN T02]? | | Yes | | | | 1 | | ***If 2, 88, 99 🡪 B04*** |
|  |  |  | No | | | | 2 | |  |
|  |  |  | *Don’t know* | | | | *88* | |  |
|  |  |  | *Refusal* | | | | *99* | |  |
|  | How many times have you been pregnant since [PREGNANCY FROM DATE IN T02]?  *Enter number of times in the space provided. If woman answers “None”, enter 00 “Don’t know”, enter 88. If woman refuses to answer, enter 99.* | | \|  \|  \| \| --- \| --- \| \| Times \| \| | | | | | |  |
| **INTERVIEWER:**  The next questions I am going to ask you refer to the pregnancy you had around [DATE FROM T02]. Though it may seem like it was a long time ago, please try to remember to the best of your ability what happened during that pregnancy.  ***(If no maternal loss to follow-up [from T03] 🡪 Section C)*** | | | | | | | | | |
|  | According to your records at [CLINIC NAME FROM T01] you have not been to [CLINIC NAME FROM T01] to receive HIV **care in the last three months**. Is this true?  By HIV care, I mean any services related to your HIV status, including regular clinic visit, pharmacy pick-up, or lab test. | | Yes | | | 1 | | | ***If 2, 88, 99 🡪 B12*** |
|  |  |  | No | | | 2 | | |  |
|  |  |  | *Don’t know* | | | *88* | | |  |
|  |  |  | *Refusal* | | | *99* | | |  |
|  | Did you stop attending HIV care at [CLINIC NAME FROM T01] before or after delivery? | | Before | | | 1 | | |  |
|  |  |  | After | | | 2 | | |  |
|  |  |  | *Don’t know* | | | *88* | | |  |
|  |  |  | *Refusal* | | | *99* | | |  |
|  | What were the reasons you stopped attending HIV care at [CLINIC NAME FROM T01]?  *Do not read options for explanation and circle all that apply.*  *If participant does not give a response, prompt by saying:*  Was it for reasons due to: Cost? Transportation? Your need for care? How you were feeling? Family obligations? Work obligations? Stigma? The quality of care? Your medication? Or other reasons? | | **Health** | | | | | | ***If 7🡪 B07***  ***All other responses 🡪 B08*** |
|  |  |  | I am no longer pregnant; didn’t think I need to go | | | 1 | | |  |
|  |  |  | I am no longer breastfeeding; didn’t think I need to go | | | 2 | | |  |
|  |  |  | I am not sure I am really HIV-positive | | | 3 | | |  |
|  |  |  | I was too tired /ill to go | | | 4 | | |  |
|  |  |  | I was feeling fine; didn’t think I needed to go | | | 5 | | |  |
|  |  |  | **Location/transportation** | | | | | |  |
|  |  |  | Clinic located too far away/travel too long (no relocation) | | | 6 | | |  |
|  |  |  | Relocated – clinic now too far away/travel too long | | | 7 | | |  |
|  |  |  | Lack of transportation to clinic | | | 8 | | |  |
|  |  |  | Transportation too costly | | | 9 | | |  |
|  |  |  | **Cost/income** | | | | | |  |
|  |  |  | Clinic services too costly | | | 10 | | |  |
|  |  |  | Job/employer will not allow me time away from work | | | 11 | | |  |
|  |  |  | **Family** | | | | | |  |
|  |  |  | Cannot arrange childcare while I visit clinic | | | 12 | | |  |
|  |  |  | Family obligations | | | 13 | | |  |
|  |  |  | Spouse/partner did not give me permission to go | | | 14 | | |  |
|  |  |  | Friend /family member told me not to go | | | 15 | | |  |
|  |  |  | **Stigma/disclosure** | | | | | |  |
|  |  |  | Not disclosed to partner – partner may discover HIV status | | | 16 | | |  |
|  |  |  | HIV status may be discovered by my family or others I know | | | 17 | | |  |
|  |  |  | **Clinic** | | | | | |  |
|  |  |  | Clinic does not have enough medications | | | 18 | | |  |
|  |  |  | Staff is not nice | | | 19 | | |  |
|  |  |  | Clinic services are not good | | | 20 | | |  |
|  |  |  | Clinic wait time is too long | | | 21 | | |  |
|  |  |  | Clinic does not offer services for mother and baby simultaneously | | | 22 | | |  |
|  |  |  | Clinic doesn’t offer the services I need (e.g., food)  Specify service needed: ___________ | | | 23 | | |  |
|  |  |  | **Medication** | | | | | |  |
|  |  |  | I never started ART so care was not helping me | | | 24 | | |  |
|  |  |  | The medication I received gave me side effects | | | 25 | | |  |
|  |  |  | The medication does not work | | | 26 | | |  |
|  |  |  | I prefer to take traditional medicines | | | 27 | | |  |
|  |  |  | **Information** | | | | | |  |
|  |  |  | I was not given an appointment to attend the clinic | | | 28 | | |  |
|  |  |  | I was referred to another clinic | | | 29 | | |  |
|  |  |  | **Other** | | | | | |  |
|  |  |  | Other 1: ______________________ | | | 30 | | |  |
|  |  |  | Other 2: ______________________ | | | 31 | | |  |
|  |  |  | Other 3: ______________________ | | | 32 | | |  |
|  |  |  | Other 4: ______________________ | | | 33 | | |  |
|  |  |  | Other 5: ______________________ | | | 34 | | |  |
|  |  |  | *Don’t know* | | | *88* | | |  |
|  |  |  | *Refusal* | | | *99* | | |  |
|  | What was or were the reasons for your relocation?  *Select all that apply.* | | Pregnancy-related: Relocated to spend my pregnancy elsewhere | | | 1 | | |  |
|  |  |  | Delivery-related : Relocated to deliver my baby elsewhere | | | 2 | | |  |
|  |  |  | Post-delivery-related: Relocated after my baby was delivered | | | 3 | | |  |
|  |  |  | Employment (self or family) | | | 4 | | |  |
|  |  |  | Other, specify: __________________ | | | 3 | | |  |
|  |  |  | *Don’t know* | | | *88* | | |  |
|  |  |  | *Refusal* | | | *99* | | |  |
|  | After you stopped going to [CLINIC IN T01] did you ever start going to an HIV clinic again (any clinic)? | | Yes | | | 1 | | | ***If 2, 88, 99🡪 B17*** |
|  |  |  | No | | | 2 | | |  |
|  |  |  | *Don’t know* | | | *88* | | |  |
|  |  |  | *Refusal* | | | *99* | | |  |
|  | Did you start going back to HIV care before or after delivery? | | Before | | | 1 | | |  |
|  |  |  | After | | | 2 | | |  |
|  |  |  | *Don’t know* | | | *88* | | |  |
|  |  |  | *Refusal* | | | *99* | | |  |
|  | Which clinic did you go back to for HIV care? | | _____________________ | | | | | | |
|  | Approximately when did you first attend HIV care at this clinic?  *If participant answers “don’t know”, probe to nearest month. If she refuses to answer, enter 99.* | | \| \|  \|  \|  \|  \|  \|  \|  \|  \| \| --- \| --- \| --- \| --- \| --- \| --- \| --- \| --- \| \|  \| **Month** \| \|  \| **Year** \| \| \| \| \| \| \| --- \| --- \| --- \| --- \| --- \| --- \| --- \| --- \| --- \| --- \| --- \| --- \| --- \| --- \| --- \| --- \| --- \| --- \| | | | | | | |
|  | How long has it been since the last time you attended the HIV clinic at [CLINIC FROM B09 or T01 if skipped from B04]?  *Enter number of months in the space provided. If woman responds a value less than one month (e.g., 3 days, or 2 weeks), enter 01 Month. If woman answers “Don’t know”, enter 88. If woman refuses to answer, enter 99.* | | \|  \|  \|  \|  \|  \|  \|  \|  \| \| --- \| --- \| --- \| --- \| --- \| --- \| --- \| --- \| \|  \| **Years** \| \|  \| **Months** \| \| \| \| \| | | | | | | |
|  | Have you attended any other HIV clinics since last attending [clinic in T01]? | | Yes | | 1 | | | | ***If 2, 88, 99🡪 B15*** |
|  |  |  | No | | 2 | | | |  |
|  |  |  | *Don’t know* | | *88* | | | |  |
|  |  |  | *Refusal* | | *99* | | | |  |
|  |  | i. At which other clinic did you receive HIV care? | ii. Approximately when did you first attend HIV care at this clinic?  *If participant answers “don’t know”, probe to nearest month. If she refuses to answer, enter 99.* | iii. When was the last time you attended HIV clinic visits at this clinic?  *If participant answers “don’t know”, probe to nearest month. If she refuses to answer, enter 99.* | | | | | |
|  | **B14a** | ________________________ | __ __ __ __ __ __  MM YYYY | __ __ __ __ __ __  MM YYYY | | | | | |
|  | **B14b** | ________________________ | __ __ __ __ __ __  MM YYYY | __ __ __ __ __ __  MM YYYY | | | | | |
|  | **B14c** | ________________________ | __ __ __ __ __ __  MM YYYY | __ __ __ __ __ __  MM YYYY | | | | | |
|  | **B14d** | ________________________ | __ __ __ __ __ __  MM YYYY | __ __ __ __ __ __  MM YYYY | | | | | |
|  | **B14e** | ________________________ | __ __ __ __ __ __  MM YYYY | __ __ __ __ __ __  MM YYYY | | | | | |
|  | **B14f** | ________________________ | __ __ __ __ __ __  MM YYYY | __ __ __ __ __ __  MM YYYY | | | | | |
|  | **B14g** | ________________________ | __ __ __ __ __ __  MM YYYY | __ __ __ __ __ __  MM YYYY | | | | | |
|  | What were the reasons you attended a different clinic than [CLINIC NAME FROM T01]?  *Do not read options for explanation and circle all that apply.*  *If participant does not give a response, prompt by saying:*  Was it for reasons due to: Cost? Transportation? Your need for care? How you were feeling? Family obligations? Work obligations? Stigma? The quality of care? Your medication? Or other reasons? | | **Location/Transportation** | | | | | | ***If 2🡪 B16***  ***All other responses 🡪 B17*** |
|  |  |  | Clinic located closer to my home /travel more reasonable (no relocation) | | | | | 1 |  |
|  |  |  | Relocated – clinic now closer | | | | | 2 |  |
|  |  |  | I have transportation to this clinic | | | | | 3 |  |
|  |  |  | **Cost/Income** | | | | | |  |
|  |  |  | Clinic services more affordable | | | | | 4 |  |
|  |  |  | Transportation more affordable | | | | | 5 |  |
|  |  |  | Job/employer will allows me time away from work to visit this clinic | | | | | 6 |  |
|  |  |  | **Family** | | | | | |  |
|  |  |  | I can arrange childcare while I visit this clinic | | | | | 7 |  |
|  |  |  | Friend /family member told me to go to this clinic | | | | | 8 |  |
|  |  |  | **Stigma/disclosure** | | | | | |  |
|  |  |  | Not disclosed to partner – partner less likely to discover HIV status at this clinic | | | | | 9 |  |
|  |  |  | HIV status less likely to be discovered by my family or others I know at this clinic | | | | | 10 |  |
|  |  |  | **Clinic** | | | | | |  |
|  |  |  | Clinic has enough medications | | | | | 11 |  |
|  |  |  | Staff are nicer | | | | | 12 |  |
|  |  |  | Clinic services are better | | | | | 13 |  |
|  |  |  | Clinic wait time is more reasonable | | | | | 14 |  |
|  |  |  | Clinic offers services for mother and baby simultaneously | | | | | 15 |  |
|  |  |  | Clinic offers the services I need (e.g., food)  *Specify service needed:* ___________ | | | | | 16 |  |
|  |  |  | **Medication** | | | | | |  |
|  |  |  | The medication from this clinic is more effective | | | | | 17 |  |
|  |  |  | **Information** | | | | | |  |
|  |  |  | I was given an appointment to attend this clinic | | | | | 18 |  |
|  |  |  | I was referred to this clinic | | | | | 19 |  |
|  |  |  | **Other** | | | | | |  |
|  |  |  | Other 1: ______________________ | | | | | 20 |  |
|  |  |  | Other 2: ______________________ | | | | | 21 |  |
|  |  |  | Other 3: ______________________ | | | | | 22 |  |
|  |  |  | Other 4: ______________________ | | | | | 23 |  |
|  |  |  | Other 5: ______________________ | | | | | 24 |  |
|  |  |  | *Don't know* | | | | | *88* |  |
|  |  |  | *Refusal* | | | | | *99* |  |
|  | What was or were the reasons for your relocation?  *Select all that apply.* | | Pregnancy-related: Relocated to spend my pregnancy elsewhere | | | | | 1 |  |
|  |  |  | Delivery-related : Relocated to deliver my baby elsewhere | | | | | 2 |  |
|  |  |  | Post-delivery-related: Relocated after my baby was delivered | | | | | 3 |  |
|  |  |  | Employment (self or family) | | | | | 2 |  |
|  |  |  | Other, specify: __________________ | | | | | 3 |  |
|  |  |  | *Don’t know* | | | | | *88* |  |
|  |  |  | *Refusal* | | | | | *99* |  |
| **INTERVIEWER:** Did any clinic visit date from B12 or B14a-g occur in the past three months? | | | Yes | | | | | 1 | ***If 1 🡪 B19***  ***If 2 🡪 B17*** |
|  |  |  | No | | | | | 2 |  |
|  | What were the reasons that you stopped attending HIV care at any clinic?  *Do not read options for explanation and circle all that apply.*  *If participant does not give a response, prompt by saying:*  Was it for reasons due to: Cost? Transportation? Your need for care? How you were feeling? Family obligations? Work obligations? Stigma? The quality of care? Your medication? Or other reasons? | | **Health** | | | | | | ***If 7🡪 B18***  ***All other responses 🡪 Section C*** |
|  |  |  | I am no longer breastfeeding; didn’t think I need to go | | | | | 1 |  |
|  |  |  | I am no longer pregnant; didn’t think I need to go | | | | | 2 |  |
|  |  |  | I am not sure I am really HIV-positive | | | | | 3 |  |
|  |  |  | I was too tired /ill to go | | | | | 4 |  |
|  |  |  | I was feeling fine; didn’t think I needed to go | | | | | 5 |  |
|  |  |  | **Location/transportation** | | | | | |  |
|  |  |  | Clinic located too far away/travel too long (no relocation) | | | | | 6 |  |
|  |  |  | Recently relocated – clinic now too far away | | | | | 7 |  |
|  |  |  | Lack of transportation to clinic | | | | | 8 |  |
|  |  |  | **Cost/Income** | | | | | |  |
|  |  |  | Transportation too costly | | | | | 9 |  |
|  |  |  | Clinic services too costly | | | | | 10 |  |
|  |  |  | Job/employer will not allow me time away from work | | | | | 11 |  |
|  |  |  | **Family** | | | | | |  |
|  |  |  | Cannot arrange childcare while I visit clinic | | | | | 12 |  |
|  |  |  | Family obligations | | | | | 13 |  |
|  |  |  | Spouse/partner did not give me permission to go | | | | | 14 |  |
|  |  |  | Friend /family member told me not to go | | | | | 15 |  |
|  |  |  | **Stigma/disclosure** | | | | | |  |
|  |  |  | Not disclosed to partner – partner may discover HIV status | | | | | 16 |  |
|  |  |  | HIV status may be discovered by my family or others I know | | | | | 17 |  |
|  |  |  | **Clinic** | | | | | |  |
|  |  |  | Clinic does not have enough medications | | | | | 18 |  |
|  |  |  | Staff is not nice | | | | | 19 |  |
|  |  |  | Clinic services are not good | | | | | 20 |  |
|  |  |  | Clinic wait time is too long | | | | | 21 |  |
|  |  |  | Clinic does not offer services for mother and baby simultaneously | | | | | 22 |  |
|  |  |  | Clinic doesn’t offer the services I need (e.g., food)  *Specify service needed:* ___________ | | | | | 23 |  |
|  |  |  | **Medication** | | | | | |  |
|  |  |  | I never started ART so care was not helping me | | | | | 24 |  |
|  |  |  | The medication I received gave me side effects | | | | | 25 |  |
|  |  |  | The medication does not work | | | | | 26 |  |
|  |  |  | I prefer to take traditional medicines | | | | | 27 |  |
|  |  |  | **Information** | | | | | |  |
|  |  |  | I was not given an appointment to attend the clinic | | | | | 28 |  |
|  |  |  | I was referred to another clinic | | | | | 29 |  |
|  |  |  | **Other** | | | | | |  |
|  |  |  | Other 1: ______________________ | | | | | 30 |  |
|  |  |  | Other 2: ______________________ | | | | | 31 |  |
|  |  |  | Other 3: ______________________ | | | | | 32 |  |
|  |  |  | Other 4: ______________________ | | | | | 33 |  |
|  |  |  | Other 5: ______________________ | | | | | 34 |  |
|  |  |  | *Don't know* | | | | | *88* |  |
|  |  |  | *Refusal* | | | | | *99* |  |
|  | What was or were the reasons for your relocation?  *Select all that apply.* | | Pregnancy-related: Relocated to spend my pregnancy elsewhere | | | | | 1 | ***All 🡪 Section C*** |
|  |  |  | Delivery-related : Relocated to deliver my baby elsewhere | | | | | 2 |  |
|  |  |  | Post-delivery-related: Relocated after my baby was delivered | | | | | 3 |  |
|  |  |  | Employment (self or family) | | | | | 2 |  |
|  |  |  | Other, specify: __________________ | | | | | 3 |  |
|  |  |  | *Don’t know* | | | | | *88* |  |
|  |  |  | *Refusal* | | | | | *99* |  |
|  | Have you taken ART in the past three months? | | Yes | | | | | 1 | ***If 2, 88, 99, 77 🡪 B24*** |
|  |  |  | No | | | | | 2 |  |
|  |  |  | *Not Applicable* | | | | | *77* |  |
|  |  |  | *Don’t Know* | | | | | *88* |  |
|  |  |  | *Refusal* | | | | | *99* |  |
|  | Have you taken ART in the past 30 days? | | Yes | | | | | 1 | ***If 2, 88, 99, 77 🡪 B24*** |
|  |  |  | No | | | | | 2 |  |
|  |  |  | *Not Applicable* | | | | | *77* |  |
|  |  |  | *Don’t Know* | | | | | *88* |  |
|  | In the last 30 days, on how many days did you miss at least one dose of any of your HIV medicines? | | No. of days: _____________ (0-30) | | | | | |  |
|  | In the last 30 days, how good a job did you do at taking your HIV medicines in the way that you were supposed to? | | Very poor | | | | | 1 |  |
|  |  |  | Poor | | | | | 2 |  |
|  |  |  | Fair | | | | | 3 |  |
|  |  |  | Good | | | | | 4 |  |
|  |  |  | Very good | | | | | 5 |  |
|  |  |  | Excellent | | | | | 6 |  |
|  |  |  | *Not Applicable* | | | | | *77* |  |
|  |  |  | *Don’t Know* | | | | | *88* |  |
|  | In the last 30 days, how often did you take your HIV medicines in the way that you were supposed to? | | Never | | | | | 1 | ***All 🡪 Section C*** |
|  |  |  | Rarely | | | | | 2 |  |
|  |  |  | Sometimes | | | | | 3 |  |
|  |  |  | Usually | | | | | 4 |  |
|  |  |  | Almost always | | | | | 5 |  |
|  |  |  | Always | | | | | 6 |  |
|  |  |  | *Not Applicable* | | | | | *77* |  |
|  |  |  | *Don’t Know* | | | | | *88* |  |
| **B24** | We are now more interested in why you stopped attending HIV care since your most recent clinic.  What were the reasons that you stopped attending HIV care at any clinic?  *Do not read options for explanation and circle all that apply.*  *If participant does not give a response, prompt by saying:*  Was it for reasons due to: the ART medicines? Your health? Transportation? Cost? Family issues? Work obligations? Stigma? The quality of care? Or other reasons? | | **ART medicines** | | | | | |  |
|  |  |  | I never started ART | | | | | 1 |  |
|  |  |  | The clinic/provider told me I was ineligible (CD4+ too high) | | | | | 2 |  |
|  |  |  | I do not have a private place to store the medication | | | | | 3 |  |
|  |  |  | The clinic did not have ART stock | | | | | 4 |  |
|  |  |  | I lost my medications | | | | | 5 |  |
|  |  |  | My medications were stolen | | | | | 6 |  |
|  |  |  | I finished my medications; ran out | | | | | 7 |  |
|  |  |  | I sold my medications | | | | | 8 |  |
|  |  |  | **Health** | | | | | |  |
|  |  |  | I am no longer breastfeeding; didn’t think I need to go | | | | | 9 |  |
|  |  |  | I am no longer pregnant; didn’t think I need to go | | | | | 10 |  |
|  |  |  | I am not sure I am really HIV-positive | | | | | 11 |  |
|  |  |  | I was too tired /ill to go | | | | | 12 |  |
|  |  |  | I was feeling fine; didn’t think I needed to go | | | | | 13 |  |
|  |  |  | **Location/transportation** | | | | | |  |
|  |  |  | Clinic located too far away/travel too long (no relocation) | | | | | 14 |  |
|  |  |  | Recently relocated – clinic now too far away | | | | | 15 |  |
|  |  |  | Lack of transportation to clinic | | | | | 16 |  |
|  |  |  | **Cost/Income** | | | | | |  |
|  |  |  | Transportation too costly | | | | | 17 |  |
|  |  |  | Clinic services or medicine too costly | | | | | 18 |  |
|  |  |  | Job/employer will not allow me time away from work | | | | | 19 |  |
|  |  |  | **Family** | | | | | |  |
|  |  |  | I did not want to tell my family members that I was taking ART | | | | | 20 |  |
|  |  |  | Cannot arrange childcare while I visit clinic | | | | | 21 |  |
|  |  |  | Family obligations | | | | | 22 |  |
|  |  |  | Spouse/partner did not give me permission to go | | | | | 23 |  |
|  |  |  | Friend /family member told me not to go | | | | | 24 |  |
|  |  |  | **Stigma/disclosure** | | | | | |  |
|  |  |  | Not disclosed to partner – partner may discover HIV status | | | | | 25 |  |
|  |  |  | HIV status may be discovered by my family or others I know | | | | | 26 |  |
|  |  |  | **Clinic** | | | | | |  |
|  |  |  | Clinic does not have enough medications | | | | | 27 |  |
|  |  |  | Staff is not nice | | | | | 28 |  |
|  |  |  | Clinic services are not good | | | | | 29 |  |
|  |  |  | Clinic wait time is too long | | | | | 30 |  |
|  |  |  | Clinic does not offer services for mother and baby simultaneously | | | | | 31 |  |
|  |  |  | Clinic doesn’t offer the services I need (e.g., food)  *Specify service needed:* ___________ | | | | | 32 |  |
|  |  |  | **Medication** | | | | | |  |
|  |  |  | I never started ART so care was not helping me | | | | | 33 |  |
|  |  |  | The medication I received gave me side effects | | | | | 34 |  |
|  |  |  | The medication does not work | | | | | 35 |  |
|  |  |  | I prefer to take traditional medicines | | | | | 36 |  |
|  |  |  | **Information** | | | | | |  |
|  |  |  | I was not given an appointment to attend the clinic | | | | | 37 |  |
|  |  |  | I was referred to another clinic | | | | | 38 |  |
|  |  |  | **Other** | | | | | |  |
|  |  |  | Other 1: ______________________ | | | | | 39 |  |
|  |  |  | Other 2: ______________________ | | | | | 40 |  |
|  |  |  | Other 3: ______________________ | | | | | 41 |  |
|  |  |  | Other 4: ______________________ | | | | | 42 |  |
|  |  |  | Other 5: ______________________ | | | | | 43 |  |
|  |  |  | *Don't know* | | | | | *88* |  |
|  |  |  | *Refusal* | | | | | *99* |  |

| **SeCTION C. INFANT CARE & HEALTH oUTCOMES**  **(*Skip to Section D if the infant is not LTF)*** | | | | | | | | | | |
| --- | --- | --- | --- | --- | --- | --- | --- | --- | --- | --- |
| **No.** | **QUESTIONS & INSTRUCTIONS** | | | **RESPONSES** | | | |  | | **skips** |
| **INTERVIEWER:** Infant health status [from T05] | | | | Alive | | | | 1 | | ***If 1, 3 🡪 C07***  ***If 2, 88, 99 🡪 C13*** |
|  |  |  |  | Infant died <6 weeks after delivery | | | | 2 | |  |
|  |  |  |  | Infant died >6 weeks after delivery | | | | 3 | |  |
|  |  |  |  | *Don’t know* | | | | *88* | |  |
|  |  |  |  | *Refusal* | | | | *99* | |  |
| **INTERVIEWER:** Now I would like to ask you some questions about your infant. Remember, these questions are in reference to the infant you were pregnant with around [DATE FROM T02]. | | | | | | | | | | |
| **C01** | Was your infant a male or female? | | | Female | | | | | 1 |  |
|  |  |  |  | Male | | | | | 2 |  |
|  |  |  |  | *Refusal* | | | | | *99* |  |
| **C02** | How much did your infant weight when s/he was born? | | | *________________ kgs* | | | | | | |
| **C03** | How old was your infant when s/he died?  *If participant answers “don’t know”, probe to nearest month. If she refuses to answer, enter ‘99 months, 99 weeks’. If the participant gives the month of birth, calculate the age and enter.* | | | \|  \|  \| \| --- \| --- \| \| Years \| \|  \|  \|  \| \| --- \| --- \| \| Months \| \| \|  \|  \| \| Weeks \| \| | | | | | | ***If 88, 99 🡪 C13*** |
| **C04** | Before she/he died, did your infant attend any visits in the ‘Under-5’ clinic? | | | Yes | | | | | 1 | ***If 1 🡪 C07***  ***If 88, 99 🡪 Section D*** |
|  |  |  |  | No | | | | | 2 |  |
|  |  |  |  | *Don’t know* | | | | | *88* |  |
|  |  |  |  | *Refusal* | | | | | *99* |  |
| **C05** | What was the reason why your infant did not attend the ‘Under-5’ clinic?  *Do not read options for explanation and circle all that apply.*  *If participant does not give a response, prompt by saying:*  Was it for reasons due to: Cost? Transportation? Your infant’s need for care? How your infant or you feeling? Family obligations? Work obligations? Stigma? The quality of care? Your infant’s medication? Or other reasons?  *.* | | | **Health** | | | | | | ***If 10 🡪 C06***  ***All other responses 🡪 C13*** |
|  |  |  |  | I was too tired /ill to take my infant | | | | | 1 |  |
|  |  |  |  | My infant was too tired/ill go to | | | | | 2 |  |
|  |  |  |  | My infant was feeling fine; didn’t think s/he needed to go | | | | | 3 |  |
|  |  |  |  | I was not breastfeeding; didn’t think I need to take him/her | | | | | 4 |  |
|  |  |  |  | I was not sure I was really HIV-positive/infant was exposed | | | | | 5 |  |
|  |  |  |  | My infant already received a final infection status; didn’t think I need to take him/her | | | | | 6 |  |
|  |  |  |  | I did not want to know my infant’s infection status | | | | | 7 |  |
|  |  |  |  | My infant has already completed all immunizations | | | | | 8 |  |
|  |  |  |  | **Location/transportation** | | | | | |  |
|  |  |  |  | Clinics were located too far away/travel too long (no relocation) | | | | | 9 |  |
|  |  |  |  | Had recently relocated – clinic was now too far away | | | | | 10 |  |
|  |  |  |  | Lack of transportation to clinics | | | | | 11 |  |
|  |  |  |  | **Cost/Income** | | | | | |  |
|  |  |  |  | Transportation too costly | | | | | 12 |  |
|  |  |  |  | Clinic services too costly | | | | | 13 |  |
|  |  |  |  | Job/employer would not allow me time away from work | | | | | 14 |  |
|  |  |  |  | **Family** | | | | | |  |
|  |  |  |  | Could not arrange childcare while I visited a clinic | | | | | 15 |  |
|  |  |  |  | Family obligations | | | | | 16 |  |
|  |  |  |  | I was not in charge of my infant’s care/infant did not live with me | | | | | 17 |  |
|  |  |  |  | Spouse/partner did not give me permission to go/ bring the baby | | | | | 18 |  |
|  |  |  |  | Friend /family member told me not to go/bring the baby | | | | | 19 |  |
|  |  |  |  | **Stigma/disclosure** | | | | | |  |
|  |  |  |  | Not disclosed to partner – partner may discover my HIV status | | | | | 20 |  |
|  |  |  |  | My HIV status may be discovered by my family or others I know | | | | | 21 |  |
|  |  |  |  | Not disclosed to partner – partner may discover my infant’s HIV exposure/status | | | | | 22 |  |
|  |  |  |  | My infant’s HIV exposure/status may be discovered by my family or others I know | | | | | 23 |  |
|  |  |  |  | **Clinic** | | | | | |  |
|  |  |  |  | Clinic did not have enough medications | | | | | 24 |  |
|  |  |  |  | Staff was not nice | | | | | 25 |  |
|  |  |  |  | Clinic services were not good | | | | | 26 |  |
|  |  |  |  | Clinic wait time was too long | | | | | 27 |  |
|  |  |  |  | Clinic did not offer services for mother and baby simultaneously | | | | | 28 |  |
|  |  |  |  | Clinic didn’t offer the services my infant/myself needs (e.g., food)  Specify service needed: ___________ | | | | | 29 |  |
|  |  |  |  | **Information** | | | | | |  |
|  |  |  |  | My infant was not given an appointment to attend the clinic | | | | | 30 |  |
|  |  |  |  | I asked for my infant be referred to another clinic but s/he was not referred | | | | | 31 |  |
|  |  |  |  | **Medication** | | | | | |  |
|  |  |  |  | The medication would have given my infant side effects | | | | | 32 |  |
|  |  |  |  | The medication the clinic would have given my infant did not work | | | | | 33 |  |
|  |  |  |  | I preferred to give my infant traditional medicines | | | | | 34 |  |
|  |  |  |  | I did not want my infant to have immunizations/immunizations do not work | | | | | 35 |  |
|  |  |  |  | **Other** | | | | | |  |
|  |  |  |  | Other 1: ______________________ | | | | | 36 |  |
|  |  |  |  | Other 2: ______________________ | | | | | 37 |  |
|  |  |  |  | Other 3: ______________________ | | | | | 38 |  |
|  |  |  |  | Other 4: ______________________ | | | | | 39 |  |
|  |  |  |  | Other 5: ______________________ | | | | | 40 |  |
|  |  |  |  | *Don’t know* | | | | *88* | |  |
|  |  |  |  | *Refusal* | | | | *99* | |  |
| **C06** | What was or were the reasons for your relocation?  *Select all that apply.* | | | Related to delivery or care of baby | | | | 1 | | ***🡪C13*** |
|  |  |  |  | Employment (self or family) | | | | 2 | |  |
|  |  |  |  | Other, specify: __________________ | | | | 3 | |  |
|  |  |  |  | *Don’t know* | | | | *88* | |  |
|  |  |  |  | *Refusal* | | | | *99* | |  |
| **C07** | How many visits did your infant attend in an ‘Under-5’ Clinic?  *Enter number of visits in the space provided. If woman answers “None”, enter 00 “Don’t know”, enter 88. If woman refuses to answer, enter 99.* | | | \|  \|  \| \| --- \| --- \| \| Visits \| \| | | | | | |  |
| **C08** | At which clinic did your infant first receive care? | | | _____________________ | | | | | | |
| **C09** | How old was your infant when s/he first received care at this clinic?  *Enter response in months. If participant answers “don’t know”, probe to nearest month. If she refuses to answer, enter ‘99 months, 99 weeks’. If the participant gives the month of birth, calculate the age and enter.* | | | \| \|  \|  \| \| --- \| --- \| \| Months \| \| \|  \|  \| \| Weeks \| \| \| \| --- \| --- \| --- \| --- \| --- \| --- \| --- \| --- \| --- \| | | | | | | |
| **C10** | How old was your infant the most recent time s/he visited the ‘Under Five’ clinic at [CLINIC FROM C08]?  *Enter response in months. If participant answers “don’t know”, probe to nearest month. If she refuses to answer, enter ‘99 months, 99 weeks’. If the participant gives the month of birth, calculate the age and enter.* | | | \|  \|  \| \| --- \| --- \| \| Months \| \| \|  \|  \| \| Weeks \| \| | | | | | | |
| **C11** | Did your infant attend another ‘Under-Five’ clinic **during her/his first 18 months?** | | | Yes | | | | | 1 | ***If 2, 88, 99 🡪 C13*** |
|  |  |  |  | No | | | | | 2 |  |
|  |  |  |  | *Don’t know* | | | | | *88* |  |
|  |  |  |  | *Refusal* | | | | | *99* |  |
| **C12** |  | i. Which other ‘Under-5’ clinics did your infant attend during his/her first 18 months? | ii. Approximately how old was your infant when s/he first attended this ‘Under-5’ clinic?  *Enter response in months. If participant answers “don’t know”, probe to nearest month. If she refuses to answer, enter ‘99 months, 99 weeks’. If the participant gives the month of birth, calculate the age and enter.* | | | iii. Approximately how old was your infant when s/he last attended this ‘Under-5’ clinic?  *Enter response in months. If participant answers “don’t know”, probe to nearest month. If she refuses to answer, enter ‘99 months, 99 weeks’. If the participant gives the month of birth, calculate the age and enter.* | | | | |
|  | **C12a** | _______________________ | __ __ __ __  MOS WKS | | | __ __ __ __  MOS WKS | | | | |
|  | **C12b** | _______________________ | __ __ __ __  MOS WKS | | | __ __ __ __  MOS WKS | | | | |
|  | **C12c** | _______________________ | __ __ __ __  MOS WKS | | | __ __ __ __  MOS WKS | | | | |
|  | **C12d** | _______________________ | __ __ __ __  MOS WKS | | | __ __ __ __  MOS WKS | | | | |
|  | **C12e** | _______________________ | __ __ __ __  MOS WKS | | | __ __ __ __  MOS WKS | | | | |
|  | **C12f** | _______________________ | __ __ __ __  MOS WKS | | | __ __ __ __  MOS WKS | | | | |
|  | **C12g** | _______________________ | __ __ __ __  MOS WKS | | | __ __ __ __  MOS WKS | | | | |
| **C13** | Did your baby receive a test for HIV at [the dates specified]?  *Ask all* | | | Around 6 weeks | *1 Yes* | | 2 No | | | ***If All 2, 88, 99 🡪 Section D*** |
|  |  |  |  | Around 6months | *1 Yes* | | 2 No | | |  |
|  |  |  |  | *9 months or later* | *1 Yes* | | *2 No* | | |  |
|  |  |  |  | *Baby tested but don’t know date* | | | *77* | | |  |
|  |  |  |  | *Don’t know* | | | *88* | | |  |
|  |  |  |  | *Refusal* | | | *99* | | |  |
| **C14** | What was the result of your infant’s most recent HIV test? | | | Reactive | | | | 1 | | ***If 2, 3, 88, 99 🡪 Section D*** |
|  |  |  |  | Non-reactive | | | | 2 | |  |
|  |  |  |  | No result/never received | | | | 3 | |  |
|  |  |  |  | Do not remember | | | | 4 | |  |
|  |  |  |  | *Don’t know* | | | | *88* | |  |
|  |  |  |  | *Refusal* | | | | *99* | |  |
| **C15** | Is your infant on ART? | | | Yes | | | | 1 | | ***If 1, 88, 99 🡪 Section D*** |
|  |  |  |  | No | | | | 2 | |  |
|  |  |  |  | *Don’t know* | | | | *88* | |  |
|  |  |  |  | *Refusal* | | | | *99* | |  |
| **C16** | Why does your infant not take ART?  *Do not read options for explanation and circle all that apply.*  *If participant does not give a response, prompt by saying:*  Is it for reasons due to: Cost? Transportation? Your infant’s need for care? How your infant or you feeling? Family obligations? Work obligations? Stigma? The quality of care? Your infant’s medication? Or other reasons? | | | **Health** | | | | | |  |
|  |  |  |  | I was too tired /ill to take my infant to the clinic | | | | 1 | |  |
|  |  |  |  | My infant was too tired/ill go to the clinic | | | | 2 | |  |
|  |  |  |  | My infant was feeling fine; didn’t think s/he needed to go | | | | 3 | |  |
|  |  |  |  | I am no longer breastfeeding; didn’t think I need to take him/her | | | | 4 | |  |
|  |  |  |  | I am not sure I am really HIV-positive/infant is exposed | | | | 5 | |  |
|  |  |  |  | My infant already received a final infection status; didn’t think I need to take him/her | | | | 6 | |  |
|  |  |  |  | I did not want to know my infant’s infection status | | | | 7 | |  |
|  |  |  |  | My infant has already completed all immunizations | | | | 8 | |  |
|  |  |  |  | **Cost/Income** | | | |  | |  |
|  |  |  |  | Medication costs too much | | | | 9 | |  |
|  |  |  |  | I sold my infant’s medications | | | | 10 | |  |
|  |  |  |  | **Family** | | | | | |  |
|  |  |  |  | I am not in charge of my infant’s care/infant does not live with me | | | | 11 | |  |
|  |  |  |  | Spouse/partner did not give me permission to start infant on ART | | | | 12 | |  |
|  |  |  |  | Friend /family member told me not to start infant on ART | | | | 13 | |  |
|  |  |  |  | **Stigma/disclosure** | | | | | |  |
|  |  |  |  | I do not have a private place to store the medication | | | | 14 | |  |
|  |  |  |  | My HIV status might be discovered | | | | 15 | |  |
|  |  |  |  | My infant’s HIV status might be discovered | | | | 16 | |  |
|  |  |  |  | I did not want to tell my family members that my infant is taking ART | | | | 17 | |  |
|  |  |  |  | I did not want to tell my spouse that my infant is taking ART | | | | 18 | |  |
|  |  |  |  | **Clinic** | | | | | |  |
|  |  |  |  | Clinic did not have enough medications | | | | 19 | |  |
|  |  |  |  | Staff was not nice | | | | 20 | |  |
|  |  |  |  | Clinic services were not good | | | | 21 | |  |
|  |  |  |  | Clinic wait time was too long | | | | 22 | |  |
|  |  |  |  | Clinic did not offer services for mother and baby simultaneously | | | | 23 | |  |
|  |  |  |  | Clinic didn’t offer the services my infant/myself needs (e.g., food)  Specify service needed: ___________ | | | | 24 | |  |
|  |  |  |  | **Information** | | | | | |  |
|  |  |  |  | The clinic/provider says my infant is ineligible | | | | 25 | |  |
|  |  |  |  | **Medication** | | | | | |  |
|  |  |  |  | The medication my infant received gave him/her side effects | | | | 26 | |  |
|  |  |  |  | The medication does not work | | | | | 27 |  |
|  |  |  |  | I prefer to give my infant traditional medicines | | | | 28 | |  |
|  |  |  |  | I lost my infant’s medications | | | | 29 | |  |
|  |  |  |  | My infant’s medications were stolen | | | | 30 | |  |
|  |  |  |  | I finished my infant’s medications; ran out | | | | 31 | |  |
|  |  |  |  | **Other** | | | | | |  |
|  |  |  |  | Other 1: ______________________ | | | | 32 | |  |
|  |  |  |  | Other 2: ______________________ | | | | 33 | |  |
|  |  |  |  | Other 3: ______________________ | | | | 34 | |  |
|  |  |  |  | Other 4: ______________________ | | | | 35 | |  |
|  |  |  |  | Other 5: ______________________ | | | | 36 | |  |
|  |  |  |  | *Don’t know* | | | | *88* | |  |
|  |  |  |  | *Refusal* | | | | *99* | |  |
| **C17** | Approximately when did your infant last take ART?  *If participant answers “don’t know”, probe to nearest month. If she refuses to answer, enter 99.* | | | Less than 1 day ago | | | | | 1 |  |
|  |  |  |  | Less than 1 week ago | | | | | 2 |  |
|  |  |  |  | Less than 1 month ago | | | | | 3 |  |
|  |  |  |  | 1-3 months ago | | | | | 4 |  |
|  |  |  |  | More than 3 months ago | | | | | 5 |  |
|  |  |  |  | *Don’t know* | | | | | *88* |  |
|  |  |  |  | *Refusal* | | | | | *99* |  |

| **SeCTION D. PATIENT CARD ABSTRACTION** | | | | | |
| --- | --- | --- | --- | --- | --- |
| **No.** | **QUESTIONS & INSTRUCTIONS** | | **RESPONSES** | | **skips** |
| *Interviewer should only review information from maternal HIV card if mom is LTF, and information from infant CWC card if infant is LTF, i.e., if patient is not LTF, then information should not be reviewed.*  **INTERVIEWER:** At this time, I would like to ask you if I could see your HIV Care Card (green card) your infant’s Child Welfare Card. I would like to collect the dates of your facility visits (and your infant’s visits, if applicable) from the cards, as well as other basic health information. Would you be willing to share them with me? | | | | | |
| **D01** | [DO NOT ASK IF INFANT DIED >6 WKS AFTER DELIVERY from T05]  Do you have your infant’s Child Welfare Card? | | Yes | 1 |  |
|  |  |  | No | 2 |  |
|  |  |  | *Refusal* | *99* |  |
|  |  |  | *Not applicable* | 77 |  |
| **D02** | Infant DOB: | | \|  \|  \|  \|  \|  \|  \|  \|  \|  \|  \| \| --- \| --- \| --- \| --- \| --- \| --- \| --- \| --- \| --- \| --- \| \| **Day** \| \|  \| **Month** \| \|  \| **Year** \| \| \| \| \| | | |
| **D03** | Infant Vaccinations: | **Visit Type** | | **Immunization Date** |  |
|  |  | **At birth** | |  |  |
|  |  | BCG | | / / |  |
|  |  | OPV 0 | | / / |  |
|  |  | **6 weeks** | |  |  |
|  |  | OPV 1 | | / / |  |
|  |  | DPT/HepB/Hib 1 | | / / |  |
|  |  | PCV 1 | | / / |  |
|  |  | **10 weeks** | |  |  |
|  |  | OPV 2 | | / / |  |
|  |  | DPT/HepB/Hib 2 | | / / |  |
|  |  | PCV 2 | | / / |  |
|  |  | **14 weeks** | |  |  |
|  |  | OPV 3 | | / / |  |
|  |  | DPT/HepB/Hib 3 | | / / |  |
|  |  | **9 months** | |  |  |
|  |  | Measles 1 | | / / |  |
|  |  | PCV 3 | | / / |  |
|  |  | **18 months** | |  |  |
|  |  | OPV 4 | | / / |  |
|  |  | Measles 2 | | / / |  |
|  |  | OPV 5 | | / / |  |
| **D04** | Infant Visits: | **Visit Type** | **Visit Date** | **DBS Barcode** | **DBS Result (R/NR/U)** |
|  |  | **0-7 days** |  |  |  |
|  |  | **7-14 days** |  |  |  |
|  |  | **6 weeks** |  |  |  |
|  |  | **10 weeks** |  |  |  |
|  |  | **14 weeks** |  |  |  |
|  |  | **6 months** |  |  |  |
|  |  | **9 months** |  |  |  |
|  |  | **12 months** |  |  |  |
|  |  | **15 months** |  |  |  |
|  |  | **18 months** |  |  |  |
|  |  | **24 months** |  |  |  |
| **D05** | Do you have your HIV Care Card (green card)? | Yes | | 1 |  |
|  |  | No | | 2 |  |
|  |  | *Refusal* | | *99* |  |
|  |  | *Not applicable* | | 77 |  |
| **D06** | Maternal HIV care visits: | **Visit Date** | | **ART Prescribed?** *Indicate ‘Y’ for ‘yes’ or ‘N’ for ‘no’.* | |
|  |  | / / | |  | |
|  |  | / / | |  | |
|  |  | / / | |  | |
|  |  | / / | |  | |
|  |  | / / | |  | |
|  |  | / / | |  | |
|  |  | / / | |  | |
|  |  | / / | |  | |
|  |  | / / | |  | |
|  |  | / / | |  | |
|  |  | / / | |  | |
|  |  | / / | |  | |
|  |  | / / | |  | |
|  |  | / / | |  | |
|  |  | / / | |  | |
|  |  | / / | |  | |
|  |  | / / | |  | |
|  |  | / / | |  | |
|  |  | / / | |  | |
|  |  | / / | |  | |
|  |  | / / | |  | |
|  |  | / / | |  | |

| **T09** | Interview stop time: | \|  \|  \| **:** \|  \|  \| \| --- \| --- \| --- \| --- \| --- \| \| **Hour** \| \|  \| **Minute** \| \| |
| --- | --- | --- | --- | --- | --- | --- | --- | --- | --- | --- | --- | --- |

| **Feedback** |
| --- |
| **QUESTIONS & INSTRUCTIONS** |
| Please provide any other comments:  ***____________________________________________________________________________________________________________________________________________________________________________________________________________________________________________________________________________________________________________________________________________________________________________________________________________________________________________________________________________________________________________________________________________________________________________________________________________*** |
