# Supplementary material for: Engagement in care among women and their infants lost to follow-up under Option B+ in eSwatini
Source: PLoS One. 2019 Oct 30;14(10):e0222959. doi: 10.1371/journal.pone.0222959 (PMC6821080; doi:10.1371/journal.pone.0222959)
Supplement: S4 Appendix — (DOCX) [file pone.0222959.s011.docx]

| **TRACING QUESTIONNAIRE**  **COMPLETE ALL OF THE INFORMATION ON THIS PAGE BEFORE BEGINNING THE INTERVIEW.** | | | |
| --- | --- | --- | --- |
|  | PMTCT enrolment clinic | Siteki PHU | 1 |
|  |  | MSF Matsapha | 2 |
|  |  | Lamvelase Clinic | 3 |
|  |  | Siphofaneni Clinic | 4 |
|  |  | RFM Hospital | 5 |
|  |  | King Sobhuza II PHU | 6 |
|  |  | Mankayane PHU | 7 |
|  |  | Luyengo PHU | 8 |
|  |  | FLAS Manzini | 9 |
|  |  | Mbikwakhe Clinic | 10 |
|  | Date of first HIV care visit: | \|  \|  \|  \|  \|  \|  \|  \|  \|  \|  \| \| --- \| --- \| --- \| --- \| --- \| --- \| --- \| --- \| --- \| --- \| \| **Day** \| \|  \| **Month** \| \|  \| **Year** \| \| \| \| \| | |
|  | Maternal or infant LTF at clinic [T01] | Maternal LTF | 1***🡪 T06*** |
|  |  | Infant LTF | 2 |
|  |  | Maternal and infant LTF | 3 |
|  | Infant delivery date  *From clinic records or Standard Tracing Form [D01 and D03] , where available* | \|  \|  \| \|  \| \|  \|  \| \|  \| \|  \|  \|  \|  \| \| --- \| --- \| --- \| --- \| --- \| --- \| --- \| --- \| --- \| --- \| --- \| --- \| --- \| --- \| \| **Day** \| \|  \| \| **Month** \| \| \|  \| \| **Year** \| \| \| \| \| | |
|  |  | No delivery | No delivery |
|  | Infant health status  *From clinic records or Standard Tracing Form [D02], where available* | Infant alive and healthy | 1 |
|  |  | Infant sick | 2 |
|  |  | Infant died during pregnancy | 3 |
|  |  | Infant died during delivery | 4 |
|  |  | Infant died <6 weeks after delivery | 5 |
|  |  | Infant died >6 weeks after delivery | 6 |
|  |  | Other: _________________ | *7* |
|  |  | *Don’t know* | *88* |
|  |  | *Refusal* | *99* |

|  | Interviewer code | \|  \|  \|  \| \| --- \| --- \| --- \| | | |
| --- | --- | --- | --- | --- | --- | --- | --- |
|  | SID NUMBER | \|  \|  \| **---** \|  \|  \|  \| \| --- \| --- \| --- \| --- \| --- \| --- \| | | |
| **T07** | Date of interview | \|  \|  \|  \|  \|  \|  \|  \|  \|  \|  \| \| --- \| --- \| --- \| --- \| --- \| --- \| --- \| --- \| --- \| --- \| \| **Day** \| \|  \| **Month** \| \|  \| **Year** \| \| \| \| \| | | |
|  | ***Prompt:*** Patient consented to participate? | Yes | 1 | ***If 2 🡪 END*** |
|  |  | No | 2 |  |
| **T08** | Interview start time: | \|  \|  \| **:** \|  \|  \| \| --- \| --- \| --- \| --- \| --- \| \| **Date** \| \|  \| **Month** \| \| | | |

**LOBUTAKO**: Siyabonga kutsi uvume kungenela lolucwaningo. Ungakhetsa kungaphendvuli leyo mibuto lotiva ngatsi awutsandzi kuyiphendvula. Ungangicela futsi kutsi ngichaze imibuto longayiva kahle. Ungayekela lokucocisana noma nini uma utiva kutsi awusafuni kuchubeka nelucwaningo. Ngicela ukhumbule kutsi timphendvulo takho titawubhaliswa ngenombolo hhayi libito lakho titawuphindze tigcineke tiyimfihlo. Libito lakho angeke livele kuleliphepha lemibuto.

| **SeCTION A. DEMOGRAPHICS** | | | | |
| --- | --- | --- | --- | --- |
| **No.** | **QUESTIONS & INSTRUCTIONS** | **RESPONSES** | | **skips** |
| **LOBUTAKO:** **Asesicale ngemibuto ya sendlini kakho?** | | | | |
|  | Ugcine kabani esikolweni?  *Do not read options aloud. Circle one response.* | Azange sengiye | 1 |  |
|  |  | Esikolweni se Primary | 2 |  |
|  |  | Ngacedza ePrimary | 3 |  |
|  |  | Ngaya e Secondary | 4 |  |
|  |  | Ngacedza eSecondary | 5 |  |
|  |  | Ngaya eHigh school | 6 |  |
|  |  | Ngacedza eHigh school | 7 |  |
|  |  | Ngafundzela kancane eKolishi | 8 |  |
|  |  | Ngacedza eKolishi | 9 |  |
|  |  | *Angati* | *88* |  |
|  |  | *Kwala* | *99* |  |
|  | Usebenta umsebenti wani kwanyalo?  *Do not read options aloud. Circle all that apply.* | Self-employed | 1 |  |
|  |  | Ngicashiwe ngiyavukela onkhe emalanga | 2 |  |
|  |  | Ngihle ngisebenta hhayi sonkhe sikhatsi | 3 |  |
|  |  | Angisebenti kwanyalo | 4 |  |
|  |  | *Angati* | *88* |  |
|  |  | *Kwala* | *99* |  |
|  | Nguyiphi indlela lengenisa imali leningi lawuhlala khona?  *Circle one response.* | Ngiyatisebenta | 1 |  |
|  |  | Ngicashiwe ngiyavukela onkhe emalanga | 2 |  |
|  |  | Ngihle ngisebenta hhayi sonkhe sikhatsi | 3 |  |
|  |  | Ngiyatsengisa angikacashwa ngalokusemtsetfweni | 4 |  |
|  |  | Imali yelabeswele | 5 |  |
|  |  | Impenisheli | 6 |  |
|  |  | Ngemiholo yalesihlala nabo endlini | 7 |  |
|  |  | Lokunye lokuphana imali, chaza:  ______________________________ | 8 |  |
|  |  | Lokunye, chaza: ______________________________ | 9 |  |
|  |  | Kute | 10 |  |
|  |  | *Angati* | *88* |  |
|  |  | *Kwala* | *99* |  |
|  | Kulabo lenihlala nabo endlini ukhona yini lonako loku lokulandzelako:    *Read the list, circle item if owned. Multiple responses are possible.* | Libhayisikili | 1 |  |
|  |  | Sidudu noma sikutha | 2 |  |
|  |  | Imoto | 3 |  |
|  |  |  |  |  |
|  |  | Liwashi | 4 |  |
|  |  | I redo | 5 |  |
|  |  | I TV | 6 |  |
|  |  | IFriji | 7 |  |
|  |  | Emasimi ekulima | 8 |  |
|  |  | Indlu | 9 |  |
|  |  | Mahlalekhikhini | 10 |  |
|  |  | Umubhedze | 11 |  |
|  |  | *Angati* | *88* |  |
|  |  | *Kwala* | *99* |  |
|  | Sewuhlale sikhatsi lesinganani kulendlu lohlala kiyo?  *Enter number of years in the space provided. If woman responds a value less than one year (e.g., 6 months), enter 01. If woman answers “Don’t know”, enter 88. If woman refuses to answer, enter 99.* | \|  \|  \| \| --- \| --- \| \| Iminyaka \| \| | |  |
|  | Bangakhi bantfu labadzala nebantfwana lohlala nabo. | \|  \|  \| \| --- \| --- \| \| Labadzala \| \| | |  |
|  |  | \|  \|  \| \| --- \| --- \| \| Bantfwana \| \| | |  |
|  | Kuletinyanga letingu 12 letengcile, ubengekho ekhaya kangakhi sikhatsi lesingaba ngetulu kwenyanga?  *Enter number of times in the space provided. If woman answers “None”, enter 00 “Don’t know”, enter 88. If woman refuses to answer, enter 99.* | \|  \|  \| \| --- \| --- \| \| Emahlandla \| \| | |  |
|  | Wake wadivosa yini noma wafelwa ngumyeni?  *Multiple responses are possible.* | Yebo ngekudivosa | 1 |  |
|  |  | Yebo ngebufelokati | 2 |  |
|  |  | Cha | 3 |  |
|  |  | *Kwala* | *99* |  |
|  | Ukhona yini lotsandzana naye? | Yebo | 1 | ***2 🡪 A13*** |
|  |  | Cha | 2 |  |
|  |  | Kwala | 99 |  |
|  | Ngabe nishadile? | Yebo | 1 |  |
|  |  | Cha | 2 |  |
|  |  | Kwala | 99 |  |
|  | Nihlala kanyekanye yini? | Yebo | 1 |  |
|  |  | Cha | 2 |  |
|  |  | Kwala | 99 |  |
|  | Senitsandzane sikhatsi lesinganani?  *Enter number of months and years in the spaces provided. If woman responds a value less than one month (e.g., 3 days, or 2 weeks), enter 01 Month and 00 Years. If woman answers “Don’t know”, enter 88 months and 88 years. If woman refuses to answer, enter 99 months and 99 years.* | \|  \|  \| \| --- \| --- \| \| Tinyanga \| \| \|  \|  \| \| Iminyaka \| \| | |  |
|  | Kukhona yini losomutjele ngesimo sakho sengati? | Yebo | 1 | ***2🡪 Section B*** |
|  |  | Cha | 2 |  |
|  |  | *Kwala* | *99* |  |
|  | Ngubani losomutjelile ngesimo sakho sengati? | *Lovana naye/ umyeni wakho* | *1* |  |
|  |  | *Lomunye lolalana naye longavani naye* | *2* |  |
|  |  | *Umtali/ Batali* | *3* |  |
|  |  | *Umntfwanakini* | *4* |  |
|  |  | *Lelinye lilunga lemndeni* | *5* |  |
|  |  | *Umngani* | *6* |  |
|  |  | *Sophumele ebaleni esiveni* | *7* |  |
|  |  | *Lokunye (Chaza)__________________* | *2* |  |
|  |  | *Kwala* | *99* |  |

| **SeCTION B. MATERNAL CARE**  ***(For all women)*** | | | | | | | | | |
| --- | --- | --- | --- | --- | --- | --- | --- | --- | --- |
| **No.** | **QUESTIONS & INSTRUCTIONS** | | **RESPONSES** | | | | | | **skips** |
| **LOBUTAKO:** Nyalo ke ngicela ku kubuta imibuto ngetemphilo yakho. Ema rekhodzi etfu akhombisa kutsi bewutetfwele nga [LUSUKU NGE T02] | | | | | | | | | |
| **LOBUTAKO** Wabeleka umntfwana lophilako yini kulokutetfwala kwakho kwaletikhatsi ta [LUSUKU NGE T02]  Refer to T04. | | | Yebo | | | | 1 | | ***If 2 🡪 B02*** |
|  |  |  | Cha | | | | 2 | |  |
|  | Wabelekela ekhaya yini noma esibhedlela? | | Wabeleka ekhaya  *Shano indzawo:*  *______________________________* | | | | 1 | |  |
|  |  |  | Wabeleka ekhaya  *Shano sibhedlela:*  *______________________________* | | | | 2 | |  |
|  |  |  | *Kwala* | | | | *99* | |  |
|  | Uke watetfwala yini kusukela nga  [LUSUKU NGE T02]? | | Yebo | | | | 1 | | ***If 2, 88, 99 🡪 B04*** |
|  |  |  | Cha | | | | 2 | |  |
|  |  |  | *Angati* | | | | *88* | |  |
|  |  |  | *Kwala* | | | | *99* | |  |
|  | Sewuke watetfwala kangakhi kusukela kuleso sikhatsi [LUSUKU NGE T02]?  *Enter number of times in the space provided. If woman answers “None”, enter 00 “Don’t know”, enter 88. If woman refuses to answer, enter 99.* | | \|  \|  \| \| --- \| --- \| \| Emahlandla \| \| | | | | | |  |
| **LOBUTAKO:**  Imibuto lelandzelako lengitakubuta yona imayelana nekutetfwala lobenako ngabo [LUSUKU NGE T02]. Noma kungabukeka ngatsi sesidze, ngiyacela uzame kukhumbula kutsi kwentekani ngesikhatsi utetfwele.  ***(If no maternal loss to follow-up [from T03] 🡪 Section C)*** | | | | | | | | | |
|  | Ngekusho kwemarekhodi akho ase [LIBITO LEMTFOLAMPHILO KU T01] awukase waya e [Libito lekliniki nge T01] kuyotfola lusito lwe HIV kuletinyanga letintsatfu letengcile. Liciniso yini leli?  Nangitsi lusito lwe HIV, ngisho lokufaka ekhatsi konkhe lodzinga kukwenta njenge kuvakashela emtfolamphilo, efasitelweni kuyolandza emaphilisi noma lapho kutsatfwa khona. | | Yebo | | | 1 | | | ***If 2, 88, 99 🡪 B12*** |
|  |  |  | Cha | | | 2 | | |  |
|  |  |  | *Angati* | | | *88* | | |  |
|  |  |  | *Kwala* | | | *99* | | |  |
|  | Wayekela kuya emtfolamphilo wase [LIBITO LEMTFOLAMPHILO KU T01] embili noma emuva kwekubeleka? | | Phambili | | | 1 | | |  |
|  |  |  | Emuva | | | 2 | | |  |
|  |  |  | *Angati* | | | *88* | | |  |
|  |  |  | *Kwala* | | | *99* | | |  |
|  | Kwaba yini sizatfu lesakwenta Wayekela kucela lusito lwekunakekelwa [LIBITO LEMTFOLAMPHILO KU T01]?  *Do not read options for explanation and circle all that apply.*  *If participant does not give a response, prompt by saying:*  Kwaba tizatfu tetindleko? Kwekuhamba? Tidzingo takho tekunakekelwa? Indlela bewutiva ngayo? Kubambeka emndenini? Kubambeka emsebentini?, kwesaba lihlazo?, lizinga lwekunakekelwa? Imitsi yakho noma lokunye? | | **Health** | | | | | | ***If 7🡪 B07***  ***All other responses 🡪 B08*** |
|  |  |  | Sengabeleka angicabanganga kutsi kusa dzingekile kutsi ngiye | | | 1 | | |  |
|  |  |  | Angisamunyisi angicabanganga kutsi kusa dzingekile kutsi ngiye | | | 2 | | |  |
|  |  |  | Angikacisiniseki kutsi vele nginalo ligciwane le HIV | | | 3 | | |  |
|  |  |  | Bengidziniwe/ngigula kakhulu kutsi ngiye | | | 4 | | |  |
|  |  |  | Bengitiva ngiphilile; angicabanganga kutsi kusa dzingekile kutsi ngiye. | | | 5 | | |  |
|  |  |  | **Location/transportation** | | | | | |  |
|  |  |  | Ikliniki ikhashane kakhulu/kuhanjwa libanga lelidze (akashintji indzawo) | | | 6 | | |  |
|  |  |  | Wahamba - Ikliniki seyikhashane kakhulu/kuhanjwa libanga lelidze | | | 7 | | |  |
|  |  |  | Kute kwekufika ekliniki | | | 8 | | |  |
|  |  |  | Kuyadula kwekuhamba kuya ekliniki | | | 9 | | |  |
|  |  |  | **Cost/income** | | | | | |  |
|  |  |  | Kudula kakhulu ekliniki | | | 10 | | |  |
|  |  |  | Emsebentini/umcashi angeke anginike sikhatsi sekuya ekliniki | | | 11 | | |  |
|  |  |  | **Family** | | | | | |  |
|  |  |  | Kute langingashiya khona umntfwana kute ngiye ekliniki | | | 12 | | |  |
|  |  |  | Kubambeka ngetintfo temndeni | | | 13 | | |  |
|  |  |  | Wakami akanginiki imvume yekuya ekliniki | | | 14 | | |  |
|  |  |  | Umngani/sihlobo singitjele kutsi ngingayi | | | 15 | | |  |
|  |  |  | **Stigma/disclosure** | | | | | |  |
|  |  |  | Wakami angikamtjeli kutsi ngiphila neligciwane – angahle abone kutsi nginalo ligciwane le HIV | | | 16 | | |  |
|  |  |  | Simo sami se HIV singahle sidaluleke emndenini wami noma  labanye lengibatiko | | | 17 | | |  |
|  |  |  | **Clinic** | | | | | |  |
|  |  |  | Ekliniki abanyo imitsi lephelele | | | 18 | | |  |
|  |  |  | Abakalungi ekliniki | | | 19 | | |  |
|  |  |  | Abasitani kahle ekliniki | | | 20 | | |  |
|  |  |  | Lidze kakhulu lilayini | | | 21 | | |  |
|  |  |  | Clinic does not offer services for mother and baby simultaneously  Abaselaphi kanye kanye neluswane | | | 22 | | |  |
|  |  |  | Abanikani tinsito lengitidzingako (njengekudla)  Chaza lokudzingako ____________ | | | 23 | | |  |
|  |  |  | **Medication** | | | | | |  |
|  |  |  | Azange ngicale kutsatsa ema ARVs ngako ke kuta lapha akungenteli lutfo | | | 24 | | |  |
|  |  |  | Lomutsi labanginika wona wawungigulisa | | | 25 | | |  |
|  |  |  | Lomutsi awusebenti | | | 26 | | |  |
|  |  |  | Nginconota kunatsa umutsi wesintfu | | | 27 | | |  |
|  |  |  | **Information** | | | | | |  |
|  |  |  | Abazange banginike lilanga lekubuya ekliniki | | | 28 | | |  |
|  |  |  | Bangilayela kulenye ikliniki | | | 29 | | |  |
|  |  |  | **Lokunye** | | | | | |  |
|  |  |  | Lokunye 1:_____________________ | | | 30 | | |  |
|  |  |  | Lokunye 2:_____________________ | | | 31 | | |  |
|  |  |  | Lokunye 3:_____________________ | | | 32 | | |  |
|  |  |  | Lokunye 4: ____________________ | | | 33 | | |  |
|  |  |  | Lokunye 5: ____________________ | | | 34 | | |  |
|  |  |  | *Angati* | | | *88* | | |  |
|  |  |  | Kwala | | | *99* | | |  |
|  | Kwabayini sizatfu lesakwenta uhambe kulendzawo?  *Select all that apply.* | | Ngenca yekutetfwala : Ngahambela kutsi ngitetfwale kulenye indzawo | | | 1 | | |  |
|  |  |  | Ngenca yekubeleka: Ngahambela kutsi ngibelekele kulenye indzawo. | | | 2 | | |  |
|  |  |  | Emuva kwekubeleka: Ngahamba ngemuva sengibelekile | | | 3 | | |  |
|  |  |  | Kucashwa (nguwe noma umndeni) | | | 4 | | |  |
|  |  |  | Lokunye, chaza:  ________________________ | | | 3 | | |  |
|  |  |  | *Angati* | | | *88* | | |  |
|  |  |  | *Kwala* | | | *99* | | |  |
|  | Uma ungasayi e [kliniki nga T01] wake wacala yini kuya kulenye ikliniki lesita nge HIV? | | Yebo | | | 1 | | | ***If 2, 88, 99🡪 B17*** |
|  |  |  | Cha | | | 2 | | |  |
|  |  |  | *Angati* | | | *88* | | |  |
|  |  |  | Kwala | | | *99* | | |  |
|  | Wabuyela ekunakekelweni ngeHIV? | | Phambi | | | 1 | | |  |
|  |  |  | ngemuva | | | 2 | | |  |
|  |  |  | *Angati* | | | *88* | | |  |
|  |  |  | *Kwala* | | | *99* | | |  |
|  | Wabuyela kuyiphi ikliniki kuyonakekelwa nge HIV? | | _____________________ | | | | | | |
|  | Nawubekisa kwaku ngunini nawucala kutotfola lusito lwe HIV kuleKliniki?  *If participant answers “don’t know”, probe to nearest month. If she refuses to answer, enter 99.* | | \| \|  \|  \|  \|  \|  \|  \|  \|  \| \| --- \| --- \| --- \| --- \| --- \| --- \| --- \| --- \| \|  \| **Inyanga** \| \|  \| **Umnyaka** \| \| \| \| \| \| \| --- \| --- \| --- \| --- \| --- \| --- \| --- \| --- \| --- \| --- \| --- \| --- \| --- \| --- \| --- \| --- \| --- \| --- \| | | | | | | |
|  | Sekubesikhatsi lesinganani solo wagcina kuya ekliniki nge HIV [CLINIC FROM B09 or T01 if skipped from B04]?  *Enter number of months in the space provided. If woman responds a value less than one month (e.g., 3 days, or 2 weeks), enter 01 Month. If woman answers “Don’t know”, enter 88. If woman refuses to answer, enter 99.* | | \|  \|  \|  \|  \|  \|  \|  \|  \| \| --- \| --- \| --- \| --- \| --- \| --- \| --- \| --- \| \|  \| **Iminyaka** \| \|  \| **Tinyanga** \| \| \| \| \| | | | | | | |
|  | Uke waya yini keleminye imitfolamphilo nge HIV solo wagcina kuta e [Kliniki le T01]? | | Yebo | | 1 | | | | ***If 2, 88, 99🡪 B15*** |
|  |  |  | Cha | | 2 | | | |  |
|  |  |  | *Angati* | | *88* | | | |  |
|  |  |  | *Kwala* | | *99* | | | |  |
|  |  | i. Kukuyiphi lenye iKliniki lapho watfola khona kunakekeleka nge HIV? | ii. Nawubekisa ucale nini kutfola lusito nge HIV kule Kliniki?  *If participant answers “don’t know”, probe to nearest month. If she refuses to answer, enter 99.* | iii. Ugcine nini kutfola lusito nge HIV kule Kliniki?  *If participant answers “don’t know”, probe to nearest month. If she refuses to answer, enter 99.* | | | | | |
|  | **B14a** | ________________________ | __ __ __ __ __ __  MM YYYY | __ __ __ __ __ __  MM YYYY | | | | | |
|  | **B14b** | ________________________ | __ __ __ __ __ __  MM YYYY | __ __ __ __ __ __  MM YYYY | | | | | |
|  | **B14c** | ________________________ | __ __ __ __ __ __  MM YYYY | __ __ __ __ __ __  MM YYYY | | | | | |
|  | **B14d** | ________________________ | __ __ __ __ __ __  MM YYYY | __ __ __ __ __ __  MM YYYY | | | | | |
|  | **B14e** | ________________________ | __ __ __ __ __ __  MM YYYY | __ __ __ __ __ __  MM YYYY | | | | | |
|  | **B14f** | ________________________ | __ __ __ __ __ __  MM YYYY | __ __ __ __ __ __  MM YYYY | | | | | |
|  | **B14g** | ________________________ | __ __ __ __ __ __  MM YYYY | __ __ __ __ __ __  MM YYYY | | | | | |
|  | Kwabayini tizatfu tekuya ekliniki leyehlukile ku [Libito le Kliniki T01]?  *Do not read options for explanation and circle all that apply.*  *If participant does not give a response, prompt by saying:*  Kwaba tizatfu temali ngenca yetindleko, tekuhamba, lokudzingako ngekugula, indlela bewutiva ngayo, kubambeka ngetintfo temndeni, kubambeka ngetintfo temsebentini, kuhlazeka, lizinga lekunakekeleka, imitsi yakho, noma letinye tizatfu? | | **Location/Transportation** | | | | | | ***If 2🡪 B16***  ***All other responses 🡪 B17*** |
|  |  |  | Yikliniki ledvutane nasekhaya/kuncono kufika khona (angikatfutsi?) | | | | | 1 |  |
|  |  |  | Ngatfutsa – sengidvute nale kliniki | | | | | 2 |  |
|  |  |  | Kukhona kwekuhamba kuleKliniki | | | | | 3 |  |
|  |  |  | **Cost/Income** | | | | | |  |
|  |  |  | Kwelashwa lapha akubiti kakhulu | | | | | 4 |  |
|  |  |  | Kwekuhamba lapha akubiti kakhulu | | | | | 5 |  |
|  |  |  | Umcashi utangivumela sikhatsi sekuya emtfolamphilo | | | | | 6 |  |
|  |  |  | **Family** | | | | | |  |
|  |  |  | Ngiyakhona kushiya umntfwana lapha nangiya kuleKlinik | | | | | 7 |  |
|  |  |  | Umngani/sihlobo sami satsi ngingayi kule kliniki | | | | | 8 |  |
|  |  |  | **Stigma/disclosure** | | | | | |  |
|  |  |  | Ngisengakamtjeli lengitsandzana naye – litfuba lekutsi akubone loko liphansi uma ngite kulekliniki | | | | | 9 |  |
|  |  |  | Kulekliniki ekhaya nalengibatiko mancane ematfuba ekutsi bati kutsi ngiphila neligciwane | | | | | 10 |  |
|  |  |  | **Clinic** | | | | | |  |
|  |  |  | Lekliniki lena inayo imitsi | | | | | 11 |  |
|  |  |  | Balunga bonesi balapha | | | | | 12 |  |
|  |  |  | Belapha ncono lapha | | | | | 13 |  |
|  |  |  | Akumiwa sikhatsi lesidze elayinini | | | | | 14 |  |
|  |  |  | Lekliniki lena iselapha sonkhe neluswane lwami | | | | | 15 |  |
|  |  |  | Lekliniki inikana nalokunye lokudzingako (njenge kudla)  Chaza lokudzingako: _____________ | | | | | 16 |  |
|  |  |  | **Medication** | | | | | |  |
|  |  |  | Imitsi yalelikliniki isebenta ngemandla kakhulu | | | | | 17 |  |
|  |  |  | **Information** | | | | | |  |
|  |  |  | Banginika lilanga lekubuya kulekliniki | | | | | 18 |  |
|  |  |  | Ngatfunyelwa betemphilo kulekliniki | | | | | 19 |  |
|  |  |  | **Lokunye** | | | | | |  |
|  |  |  | Lokunye 1:_____________________ | | | | | 20 |  |
|  |  |  | Lokunye 2:_____________________ | | | | | 21 |  |
|  |  |  | Lokunye 3:_____________________ | | | | | 22 |  |
|  |  |  | Lokunye 4: ____________________ | | | | | 23 |  |
|  |  |  | Lokunye 5: ____________________ | | | | | 24 |  |
|  |  |  | *Angati* | | | | | *88* |  |
|  |  |  | Kwala | | | | | *99* |  |
|  | Kwaba yini sizatfu lesakwenta watfutsa?  *Select all that apply.* | | Ngenca yekutetfwala: Ngatfutsa ngoba ngifuna kutsi nangitetfwele ngibe kulenye indzawo. | | | | | 1 |  |
|  |  |  | Ngenca yekubeleka: ngesuka ngoba ngifuna kubelekela kulenye indzawo | | | | | 2 |  |
|  |  |  | Emuva kwekubeleka: Ngesuka ngoba besengibelekile | | | | | 3 |  |
|  |  |  | Ngenca yekucashwa kwami noma basekhaya | | | | | 2 |  |
|  |  |  | Lokunye, chaza __________________ | | | | | 3 |  |
|  |  |  | *Angati* | | | | | *88* |  |
|  |  |  | *Kwala* | | | | | *99* |  |
| **LOBUTAKO:** Bewufanele yini ubuyele ekliniki emkhatsini we B12 or B14a-g kwenteke kuletinyanga letintsatfu letengcile? | | | Yebo | | | | | 1 | ***If 1 🡪 B19***  ***If 2 🡪 B17*** |
|  |  |  | Cha | | | | | 2 |  |
|  | Wentiwa yini kutsi ungasayi nje nhlobo emitfolamphilo uyotfola lusito nge HIV?  *Do not read options for explanation and circle all that apply.*  *If participant does not give a response, prompt by saying:*  Kungaba yini tizatfu letimayelana ne: ngenca yetindleko, tekuhamba, lokudzingako ngekugula, indlela lebe utiva ngayo kubambeka ngetintfo temndeni, kubambeka ngetintfo temsebentini, kuhlazeka, lizinga lekunakekeleka, imitsi yakho, noma letinye tizatfu | | **Health** | | | | | | ***If 7🡪 B18***  ***All other responses 🡪 Section C*** |
|  |  |  | Angisamunyisi, angizange ngicabange kutsi kusedzingekile ngiye ekliniki | | | | | 1 |  |
|  |  |  | Angisekatetfwali, angizange ngicabange kutsi kusedzingekile ngiye ekliniki | | | | | 2 |  |
|  |  |  | Angikaciniseki kutsi vele nginalo ligciwane le HIV | | | | | 3 |  |
|  |  |  | Bengidziniwe kakhulu/ngigula kutsi ngiye | | | | | 4 |  |
|  |  |  | Bengitiva ngincono; angizange ngicabange kutsi kusedzingekile ngiye ekliniki | | | | | 5 |  |
|  |  |  | **Location/transportation** | | | | | |  |
|  |  |  | Umtfolamphilo ukhashane kakhulu/ kuhanjwa sikhatsi lesidze (nakute kusuka endzaweni) | | | | | 6 |  |
|  |  |  | Ngitfutsile: ikliniki seyikhashane kakhulu | | | | | 7 |  |
|  |  |  | Kute kwekuya ekliniki | | | | | 8 |  |
|  |  |  | **Cost/Income** | | | | | |  |
|  |  |  | Kudula kakhulu kwekuhamba | | | | | 9 |  |
|  |  |  | Kwekusita kudula kakhulu ekliniki | | | | | 10 |  |
|  |  |  | Emsebentini angeke banginike sikhatsi sekuya ekliniki | | | | | 11 |  |
|  |  |  | **Family** | | | | | |  |
|  |  |  | Kute longangisalela nemntfwana kute ngiye ekliniki | | | | | 12 |  |
|  |  |  | Kubambeka ngetintfo temndeni | | | | | 13 |  |
|  |  |  | Lowakami azange angivumele ngiye | | | | | 14 |  |
|  |  |  | Umngani/sihlobo sami satsi ngingayi | | | | | 15 |  |
|  |  |  | **Stigma/disclosure** | | | | | |  |
|  |  |  | Ngisengakamtjeli lengitsandzana naye – litfuba lekutsi akubone loko liphansi uma ngite kulekliniki | | | | | 16 |  |
|  |  |  | Kulekliniki, ekhaya nalengibatiko mancane ematfuba ekutsi bati kutsi ngiphila neligciwane | | | | | 17 |  |
|  |  |  | **Clinic** | | | | | |  |
|  |  |  | Lekliniki lena inayo imitsi | | | | | 18 |  |
|  |  |  | Balunga bonesi balapha | | | | | 19 |  |
|  |  |  | Belapha ncono lapha | | | | | 20 |  |
|  |  |  | Akumiwa sikhatsi lesidze elayinini | | | | | 21 |  |
|  |  |  | Lekliniki lena iselapha sonkhe neluswane lwami | | | | | 22 |  |
|  |  |  | Lekliniki inikana nalokunye lokudzingako (njenge kudla)  Chaza lokudzingako: _____________ | | | | | 23 |  |
|  |  |  | **Medication** | | | | | |  |
|  |  |  | Azange sengiwacale ema ARVs solusito belungeke lungisite mine | | | | | 24 |  |
|  |  |  | Lomutsi lenganikwa wona wawungigulisa | | | | | 25 |  |
|  |  |  | Lemitsi ayisebenti | | | | | 26 |  |
|  |  |  | Nginconota imitsi yesintfu | | | | | 27 |  |
|  |  |  | **Information** | | | | | |  |
|  |  |  | Azange banginike lilanga kutsi ngibobuya nini | | | | | 28 |  |
|  |  |  | Kwatsiwa asale ngiya kulenye ikliniki | | | | | 29 |  |
|  |  |  | **Lokunye** | | | | | |  |
|  |  |  | Lokunye 1:_____________________ | | | | | 30 |  |
|  |  |  | Lokunye 2:_____________________ | | | | | 31 |  |
|  |  |  | Lokunye 3:_____________________ | | | | | 32 |  |
|  |  |  | Lokunye 4: ____________________ | | | | | 33 |  |
|  |  |  | Lokunye 5: ____________________ | | | | | 34 |  |
|  |  |  | *Angati* | | | | | *88* |  |
|  |  |  | *Kwala* | | | | | *99* |  |
|  | *Select all that apply.*  Kwaba yini sizatfu noma tizatfu letakwenta utfutse? | | Ngenca yekutetfwala: Ngatfutsa ngoba ngifuna kutsi nangitetfwele ngibe kulenye indzawo. | | | | | 1 | ***All 🡪 Section C*** |
|  |  |  | Ngenca yekubeleka: ngesuka ngoba ngifuna kubelekela kulenye indzawo | | | | | 2 |  |
|  |  |  | Emuva kwekubeleka: Ngesuka ngoba besengibelekile | | | | | 3 |  |
|  |  |  | Emuva kwekubeleka: Ngesuka ngoba besengibelekile | | | | | 2 |  |
|  |  |  | Lokunye, chaza: _____________ | | | | | 3 |  |
|  |  |  | *Angati* | | | | | *88* |  |
|  |  |  | *Kwala* | | | | | *99* |  |
|  | Ukewawatsatsa yini ema ARVs kuletinyanga letintsatfu letengcile? | | Yebo | | | | | 1 | ***If 1, 88, 99, 77 🡪 Section C*** |
|  |  |  | Cha | | | | | 2 |  |
|  |  |  | *Akungeni* | | | | | *77* |  |
|  |  |  | Angati | | | | | *88* |  |
|  |  |  | Kwala | | | | | *99* |  |
|  | Uke wawanatsa yini ema ARV Kulamalanga langemashumi lamatsatfu (30) lengcile. | | Yebo | | | | | 1 | ***If 2, 88, 99, 77 🡪 B24*** |
|  |  |  | Cha | | | | | 2 |  |
|  |  |  | *Akungeni* | | | | | *77* |  |
|  |  |  | *Angati* | | | | | *88* |  |
|  | Kulamalanga langemashumi lamatsatfu (30) lengcile ukewaweca yini kanye noma muphi umutsi lonikwe yona ye HIV? | | Inombolo yemalanga __________(0-30) | | | | | |  |
|  | Kulamalanga langemashumi lamatsatfu (30) lengcile uwanatse kahle kanganani ema ARVs akho kulendlela lowafundziswa kutsi kufanele ngayo? | | Kabi kakhulu | | | | | 1 |  |
|  |  |  | kabi | | | | | 2 |  |
|  |  |  | Ngalokwenetisako | | | | | 3 |  |
|  |  |  | Kahle | | | | | 4 |  |
|  |  |  | Kahle kakhulu | | | | | 5 |  |
|  |  |  | Kahle kakhulu ngalokwengcile | | | | | 6 |  |
|  |  |  | *Akungeni* | | | | | *77* |  |
|  |  |  | *Angati* | | | | | *88* |  |
|  | Kulamalanga langemashumi lamatsatfu (30) lengcile, uwatsatse kangakhi ema ARVs akho kulendlela lebewufanele uwatsatse ngayo? | | Azange | | | | | 1 | ***All 🡪 Section C*** |
|  |  |  | Bekungaka vami | | | | | 2 |  |
|  |  |  | Ngalesinye sikhatsi | | | | | 3 |  |
|  |  |  | Bekuyimvama | | | | | 4 |  |
|  |  |  | Cishe sonkhe sikhatsi | | | | | 5 |  |
|  |  |  | Sonkhe sikhatsi | | | | | 6 |  |
|  |  |  | *Akungeni* | | | | | *77* |  |
|  |  |  | *Angati* | | | | | *88* |  |
| **B24** | Kwanyalo Sifuna kuvisisa kutsi wayekelelani kuya ekliniki uyotfola lusito nge HIV kusukela kule lowagcina ngayo.  Kwaba yini sizatfu ungasayi nhlobo nje ekliniki uyotfola lusito nge HIV?  *Do not read options for explanation and circle all that apply.*  *If participant does not give a response, prompt by saying:*  Kungaba yini sizatfu lesimayelana ne: nalemitsi ema ARVs? Imphilo yakho? Kwekuhamba? Tindleko? Tintfo tasemndenini? Tintfo tasemsebentini? Lihlazo? Lizinga lwekunakekelwa? Noma letinye tizatfu? | | **ART medicines** | | | | | |  |
|  |  |  | Azange sengiwacale ema ARVs | | | | | 1 |  |
|  |  |  | Emtfolamphilo batsi emasotja ami asasetulu angikafiki ezingeni lekucala ema ARVs (CD4+ too high) | | | | | 2 |  |
|  |  |  | Ngite indzawo lesitsele langinga wabeka khona ema ARVs | | | | | 3 |  |
|  |  |  | Ekliniki abephelile ema ARVs esitokweni | | | | | 4 |  |
|  |  |  | Wangilahlekela umutsi wami (ema ARVs?) | | | | | 5 |  |
|  |  |  | Baweba umutsi wami (ema ARVs?) | | | | | 6 |  |
|  |  |  | Ngawucedza umutsi wami; aphela | | | | | 7 |  |
|  |  |  | Ngawutsengisa umutsi wami | | | | | 8 |  |
|  |  |  | **Health** | | | | | |  |
|  |  |  | Angisamunyisi; azange ngicabange kutsi kusedzingekile ngibuyele ekliniki | | | | | 9 |  |
|  |  |  | Angisekatetfwali; azange ngicabange kutsi kusedzingekile ngibuyele ekliniki | | | | | 10 |  |
|  |  |  | Angikaciniseki kutsi nginayo iHIV | | | | | 11 |  |
|  |  |  | Besengidziniwe kakhulu/ngigula kakhulu kubuyela | | | | | 12 |  |
|  |  |  | Besengitiva ngincono; azange ngicabange kutsi kusedzingekile ngibuyele ekliniki | | | | | 13 |  |
|  |  |  | **Location/transportation** | | | | | |  |
|  |  |  | Ikhashane kakhulu ikliniki/ kuhanjwa libanga lelidze kakhulu | | | | | 14 |  |
|  |  |  | Ngitfutsile – ikliniki seyikhashane kakhulu | | | | | 15 |  |
|  |  |  | Kubete kwekuya eklinikhi | | | | | 16 |  |
|  |  |  | **Cost/Income** | | | | | |  |
|  |  |  | Kudula kakhulu kwekuya ekliniki | | | | | 17 |  |
|  |  |  | Kudula kakhulu kwelashwa nemitsi | | | | | 18 |  |
|  |  |  | Angeke emsebentini/Umcashi angivumele sikhatsi ngibe ngekho emsebentini | | | | | 19 |  |
|  |  |  | **Family** | | | | | |  |
|  |  |  | Bengikafisi kutjela umndeni wami kutsi nginatsa ema ARVs | | | | | 20 |  |
|  |  |  | Kute lapho ngingashiya khona umntfwana kute ngikwati kuya ekliniki | | | | | 21 |  |
|  |  |  | Kubambeka ngemndeni | | | | | 22 |  |
|  |  |  | Wakami/lengitsandzana naye azange avume kutsi ngiye | | | | | 23 |  |
|  |  |  | Umngani/sihlobo satsi ngingayi | | | | | 24 |  |
|  |  |  | **Stigma/disclosure** | | | | | |  |
|  |  |  | Angikamatisi lengitsandzana naye simo sami sengati– angahle adalule kutsi simo sami sengati sinjani | | | | | 25 |  |
|  |  |  | Singahle sidaluleke simo sami sengati ekhaya nakulabanye lengibatiko | | | | | 26 |  |
|  |  |  | **Clinic** | | | | | |  |
|  |  |  | Ikliniki ite imitsi leyenele | | | | | 27 |  |
|  |  |  | Abakalungi labasebenta khona | | | | | 28 |  |
|  |  |  | Lusito lwasekliniki aluluhle | | | | | 29 |  |
|  |  |  | Lidze kakhulu lilayini ekliniki | | | | | 30 |  |
|  |  |  | Aselashwa kanye kanye neluswane lwami | | | | | 31 |  |
|  |  |  | *Specify service needed:*  Ekliniki abasiniki lesikweswele (e.g. kudla) | | | | | 32 |  |
|  |  |  | **Medication** | | | | | |  |
|  |  |  | Azange sengiwacale ema ARVs lusito belungangenteli lutfo | | | | | 33 |  |
|  |  |  | Lomutsi lengawutfola bewungigulisa | | | | | 34 |  |
|  |  |  | Lomutsi awusebenti | | | | | 35 |  |
|  |  |  | Nginconota imitsi yesintfu | | | | | 36 |  |
|  |  |  | **Information** | | | | | |  |
|  |  |  | Azange ngitjelwe lusuku lwekubuyela ekliniki | | | | | 37 |  |
|  |  |  | Ngatfunyelwa kulenye ikliniki | | | | | 38 |  |
|  |  |  | **Lokunye** | | | | | |  |
|  |  |  | Lokunye 1:_____________________ | | | | | 39 |  |
|  |  |  | Lokunye 2:_____________________ | | | | | 40 |  |
|  |  |  | Lokunye 3:_____________________ | | | | | 41 |  |
|  |  |  | Lokunye 4: ____________________ | | | | | 42 |  |
|  |  |  | Lokunye 5: ____________________ | | | | | 43 |  |
|  |  |  | *Angati* | | | | | *88* |  |
|  |  |  | *Kwala* | | | | | *99* |  |

| **SeCTION C. INFANT CARE & HEALTH oUTCOMES**  **(*Skip to Section D if the infant is not LTF)*** | | | | | | | | |
| --- | --- | --- | --- | --- | --- | --- | --- | --- |
| **No.** | **QUESTIONS & INSTRUCTIONS** | | | **RESPONSES** | | |  | **skips** |
| **LOBUTAKO:** Simo semphilo yeluswane [Ku T05] | | | | Uyaphila | | | 1 | ***If 1, 3 🡪 C07***  ***If 2, 88, 99 🡪 C13*** |
|  |  |  |  | Washona<6 emaviki abelekiwe | | | 2 |  |
|  |  |  |  | Washona >6 emaviki abelekiwe | | | 3 |  |
|  |  |  |  | *Angati* | | | *88* |  |
|  |  |  |  | *Kwala* | | | *99* |  |
| **LOBUTAKO:** Nyalo ke ngicela kukubuta imibuto mayelana neluswane lwakho. Ukhumbule, lemibuto imayelana nalo luswane lwakho bewulwetfwele nga [lusuku ku T02] | | | | | | | | |
| **C01** | Abengumtfwana wemfana noma wentfombatana? | | | Intfombatana | | | 1 |  |
|  |  |  |  | Umfana | | | 2 |  |
|  |  |  |  | *Kwala* | | | *99* |  |
| **C02** | Abetsini esikalini umntfwana wakho nakabelekwa? | | | *________________ Sikali* | | | | |
| **C03** | Abenganani umntfwana wakho nakashona?  *If participant answers “don’t know”, probe to nearest month. If she refuses to answer, enter ‘99 months, 99 weeks’. If the participant gives the month of birth, calculate the age and enter.* | | | \|  \|  \| \| --- \| --- \| \| Iminyaka \| \|  \|  \|  \| \| --- \| --- \| \| Tinyanga \| \| \|  \|  \| \| emaviki \| \| | | | | ***If 88, 99 🡪 C13*** |
| **C04** | Angakashoni, abeya yini emtfolamphilo walaba labangaphansi kwe minyaka lesihlanu? | | | Yebo | | | 1 | ***If 1 🡪 C07***  ***If 88, 99 🡪 Section D*** |
|  |  |  |  | Cha | | | 2 |  |
|  |  |  |  | *Angati* | | | *88* |  |
|  |  |  |  | *Kwala* | | | *99* |  |
| **C05** | Kwabayini umntfwana wakho angayi ekliniki yalaba labangaphansi kwa 5?  *Do not read options for explanation and circle all that apply.*  *If participant does not give a response, prompt by saying:*  Kungaba yini tizatfu letimayelana ne: tindleko? Kwekuhamba? Tidzingo tekunakekelwa kweluswane lwakho? Lebelutiva ngakhona luswane lwakho. Kubambeka emndenini. Kubambeka emsebentini? Lihlazo? Lizinga lwekunakekelwa? Imitsi yeluswane noma letinye tizatfu?  *.* | | | **Health** | | | | ***If 10 🡪 C06***  ***All other responses 🡪 C13*** |
|  |  |  |  | Besengidziniwe kakhulu/ngigula kakhulu kulumikisa luswane | | | 1 |  |
|  |  |  |  | Beseludziniwe kakhulu/lugula kakhulu kulumikisa luswane | | | 2 |  |
|  |  |  |  | Beluphilile luswane; azange ngicabange kutsi kudzingekile luye ekliniki | | | 3 |  |
|  |  |  |  | Bese ngingasamunyisi; azange ngicabange kutsi kudzingekile luye ekliniki | | | 4 |  |
|  |  |  |  | Bengi ngaka ciniseki kutsi nginayo iHIV/luswane lwatseleleka | | | 5 |  |
|  |  |  |  | Bese satisiwe ngalokuphelele simo sengati seluswane azange ngicabange kutsi kudzingekile luye ekliniki | | | 6 |  |
|  |  |  |  | Azange ngifune kwati simo sengati seluswane | | | 7 |  |
|  |  |  |  | Luswane lwami selujove yonkhe imijovo yalo | | | 8 |  |
|  |  |  |  | **Location/transportation** | | | |  |
|  |  |  |  | Emakliniki akhashane kakhulu kuhanjwa libanga lelidze (akatfutsi) | | | 9 |  |
|  |  |  |  | Ngatfutsa – ikliniki bese ikhashane kakhulu | | | 10 |  |
|  |  |  |  | Kwabate kwekuya emakliniki | | | 11 |  |
|  |  |  |  | **Cost/Income** | | | |  |
|  |  |  |  | Kudula kakhulu kwekuhamba | | | 12 |  |
|  |  |  |  | Kudula kakhulu ekliniki | | | 13 |  |
|  |  |  |  | Emsebentini/Umcashi abengavumi nesikhatsi ngishiye emsebentini | | | 14 |  |
|  |  |  |  | **Family** | | | |  |
|  |  |  |  | Kwabate lapho ngishiye khona Bantfwana kute ngiye ekliniki | | | 15 |  |
|  |  |  |  | Kubambeka ekhaya | | | 16 |  |
|  |  |  |  | Abengekho kimi umntfwana/ kwelapheka kwakhe kwaku ngekho etandleni tami. | | | 17 |  |
|  |  |  |  | Lengitsandzana naye azange avume ngimikise luswane ekliniki | | | 18 |  |
|  |  |  |  | Umngani/sihlobo satsi ngingamu mikisi umntfwana | | | 19 |  |
|  |  |  |  | **Stimga/disclosure** | | | |  |
|  |  |  |  | Lengitsandzana naye angikamtjeli ngesimo sami sengati – angahle abone | | | 20 |  |
|  |  |  |  | Singahle sidaluleke simo sami sengati ekhaya nakulabanye lengibatiko | | | 21 |  |
|  |  |  |  | Lengitsandzana naye angikamtjeli ngesimo sami sengati – angahle abone simo sengati yeluswane | | | 22 |  |
|  |  |  |  | Singahle sidaluleke simo sengati yeluswane ekhaya nakulabanye lengibatiko | | | 23 |  |
|  |  |  |  | **Clinic** | | | |  |
|  |  |  |  | Ekliniki bebate yonkhe imitsi | | | 24 |  |
|  |  |  |  | Beba ngakalungi emtfolamphilo | | | 25 |  |
|  |  |  |  | Belungasilo loluhle lusito lwekliniki | | | 26 |  |
|  |  |  |  | Sikhatsi sekulindza ekliniki besisidze kakhulu | | | 27 |  |
|  |  |  |  | bebangasitani kumake neluswane kanye kanye ekliniki | | | 28 |  |
|  |  |  |  | Ekliniki bebanganiketi lolusito luswane/nami besiludzinga (e.g. kudla) | | | 29 |  |
|  |  |  |  | **Information** | | | |  |
|  |  |  |  | Ekliniki Abazange basinike lusuku lwekubuya | | | 30 |  |
|  |  |  |  | Ngacela kutsi luswane lwami lushintjelwe kulenye ikliniki bala kulushintja | | | 31 |  |
|  |  |  |  | **Medication** | | | |  |
|  |  |  |  | Lemitsi beyi talugulisa luswane | | | 32 |  |
|  |  |  |  | Bebatanginike imitsi ingete yasebenta | | | 33 |  |
|  |  |  |  | Ngakhetsa kunika luswane lwami imitsi yesintfu | | | 34 |  |
|  |  |  |  | Azange ngifune luswane lwami lugome/akusebenti kugoma | | | 35 |  |
|  |  |  |  | **Lokunye** | | | |  |
|  |  |  |  | Lokunye 1:_____________________ | | | 36 |  |
|  |  |  |  | Lokunye 2:_____________________ | | | 37 |  |
|  |  |  |  | Lokunye 3:_____________________ | | | 38 |  |
|  |  |  |  | Lokunye 4: ____________________ | | | 39 |  |
|  |  |  |  | Lokunye 5: ____________________ | | | 40 |  |
|  |  |  |  | *Angati* | | | *88* |  |
|  |  |  |  | *Kwala* | | | *99* |  |
| **C06** | Wentiwa yini kutfutsa?  *Select all that apply.* | | | Lokuhambisana nekubelekwa neku nakekelwa kweluswane | | | 1 | ***🡪C13*** |
|  |  |  |  | Kucashwa (ngimi noma umndeni) | | | 2 |  |
|  |  |  |  | Lokunye chaza:  __________________ | | | 3 |  |
|  |  |  |  | *Angati* | | | *88* |  |
|  |  |  |  | *Kwala* | | | *99* |  |
| **C07** | Luye kangakhi luswane emtfolamphilo walaba phansi kweminyaka lesihlanu?  *Enter number of visits in the space provided. If woman answers “None”, enter 00 “Don’t know”, enter 88. If woman refuses to answer, enter 99.* | | | \|  \|  \| \| --- \| --- \| \| kuvakasha \| \| | | | |  |
| **C08** | Lwacala kuyiphi ikliniki luswane lwakho kunakekelwa? | | | _____________________ | | | | |
| **C09** | Lwalunganani nalucala kunakekelwa kulekliniki luswane lwakho?  *Enter response in months. If participant answers “don’t know”, probe to nearest month. If she refuses to answer, enter ‘99 months, 99 weeks’. If the participant gives the month of birth, calculate the age and enter.* | | | \| \|  \|  \| \| --- \| --- \| \| Tinyanga \| \| \|  \|  \| \| emaviki \| \| \| \| --- \| --- \| --- \| --- \| --- \| --- \| --- \| --- \| --- \| | | | | |
| **C10** | Belunganani luswane lwakho nalugcina kuya ekliniki yalaba phansi kweminyaka lesihlanu [ikliniki ku C08]  *Enter response in months. If participant answers “don’t know”, probe to nearest month. If she refuses to answer, enter ‘99 months, 99 weeks’. If the participant gives the month of birth, calculate the age and enter.* | | | \|  \|  \| \| --- \| --- \| \| tinyanga \| \| \|  \|  \| \| emaviki \| \| | | | | |
| **C11** | Beluya kulenye yini ikliniki yalaba phansi kweminyaka lesihlanu etinyangeni **tekucala letingu 18 atelwe.** | | | Yebo | | | 1 | ***If 2, 88, 99 🡪 C13*** |
|  |  |  |  | Cha | | | 2 |  |
|  |  |  |  | *Angati* | | | *88* |  |
|  |  |  |  | Kwati | | | *99* |  |
| **C12** |  | i. Ngu yiphi lenye ikliniki yalaba phansi kweminyaka lesihlanu labeya kiyo? | ii. Abenganani nawubekisa uma aya kwekucala ekliniki yalaba phansi kweminyaka lesihlanu  *Enter response in months. If participant answers “don’t know”, probe to nearest month. If she refuses to answer, enter ‘99 months, 99 weeks’. If the participant gives the month of birth, calculate the age and enter.* | | | iii. Abenganani nawubekisa uma aya kwekugcina ekliniki yalaba phansi kweminyaka lesihlanu  *Enter response in months. If participant answers “don’t know”, probe to nearest month. If she refuses to answer, enter ‘99 months, 99 weeks’. If the participant gives the month of birth, calculate the age and enter.* | | |
|  | **C12a** | _______________________ | __ __ __ __  MOS WKS | | | __ __ __ __  MOS WKS | | |
|  | **C12b** | _______________________ | __ __ __ __  MOS WKS | | | __ __ __ __  MOS WKS | | |
|  | **C12c** | _______________________ | __ __ __ __  MOS WKS | | | __ __ __ __  MOS WKS | | |
|  | **C12d** | _______________________ | __ __ __ __  MOS WKS | | | __ __ __ __  MOS WKS | | |
|  | **C12e** | _______________________ | __ __ __ __  MOS WKS | | | __ __ __ __  MOS WKS | | |
|  | **C12f** | _______________________ | __ __ __ __  MOS WKS | | | __ __ __ __  MOS WKS | | |
|  | **C12g** | _______________________ | __ __ __ __  MOS WKS | | | __ __ __ __  MOS WKS | | |
| **C13** | ? Uke wahlolwa yini umntfwana simo sengati ku [Letinsuku letichaziwe] | | | Alakubo 6 weeks | *1 Yebo* | | 2 Cha | ***If 2, 88, 99 🡪 Section D*** |
|  |  |  |  | Alakubo 6 months | *1 yebo* | | 2 Cha |  |
|  |  |  |  | *Alakubo 9 months kuya etulu* | *1 Yebo* | | *2 Cha* |  |
|  |  |  |  | *Wahlolwa kodvwa lusuku angilati* | | | *77* |  |
|  |  |  |  | *Angati* | | | *88* |  |
|  |  |  |  | *Kwala* | | | *99* |  |
|  |  | | | \|  \|  \| \| --- \| --- \| \|  \| \| | | | |  |
| **C14** | Yatsini imiphumela yalokuhlolwa ligciwane? | | | ikhona | | | 1 | ***If 2, 3, 88, 99 🡪 Section D*** |
|  |  |  |  | ayikho | | | 2 |  |
|  |  |  |  | Azange seyentiwe/kute imiphumela | | | 3 |  |
|  |  |  |  | Angikhumbuli | | | 4 |  |
|  |  |  |  | *Angati* | | | *88* |  |
|  |  |  |  | *Kwala* | | | *99* |  |
| **C15** | Is your infant on ART?  Luswane lwakho luyawatsatsa yini ema ARV | | | Yebo | | | 1 | ***If 1, 88, 99 🡪 Section D*** |
|  |  |  |  | Cha | | | 2 |  |
|  |  |  |  | *Angati* | | | *88* |  |
|  |  |  |  | *Kwala* | | | *99* |  |
| **C16** | Aluwatsatsi ngani ema ARVs luswane lwakho?  *Do not read options for explanation and circle all that apply.*  *If participant does not give a response, prompt by saying:*  Kungaba yini tizatfu letimayelana ne: tindleko? Kwekuhamba? Tidzingo tekunakekelwa kweluswane lwakho? Lebelutiva ngakhona luswane lwakho. Kubambeka emndenini. Kubambeka emsebentini? Lihlazo? Lizinga lwekunakekelwa? Imitsi yeluswane noma letinye tizatfu? | | | **Health** | | | |  |
|  |  |  |  | Besengidziniwe kakhulu/ngigula kumikisa luswane ekliniki | | | 1 |  |
|  |  |  |  | Luswane beseludziniwe kakhulu/lugula kakhulu kumikiswa ekliniki | | | 2 |  |
|  |  |  |  | Beluphilile luswane azange ngicabange kutsi kudzingekile luye ekliniki | | | 3 |  |
|  |  |  |  | Bese ngingasamunyisi; azange ngicabange kutsi kudzingekile luye ekliniki | | | 4 |  |
|  |  |  |  | Anginaso siciniseko kutsi nginalo ligciwane leHIV/luswane lwesulelekile | | | 5 |  |
|  |  |  |  | Bese satisiwe ngalokuphelele simo sengati seluswane azange ngicabange kutsi kudzingekile luye ekliniki | | | 6 |  |
|  |  |  |  | Bengingafuni kwati simo sengati yeluswane lwami | | | 7 |  |
|  |  |  |  | Luswane lwami lwayicedza imijovo yalo | | | 8 |  |
|  |  |  |  | **Cost/Income** | | |  |  |
|  |  |  |  | Imitsi ibita kakhulu | | | 9 |  |
|  |  |  |  | Ngayitsengisa imitsi yeluswane lwami | | | 10 |  |
|  |  |  |  | **Family** | | | |  |
|  |  |  |  | Alukaphatfwa ngimi luswane/aluhlali nami | | | 11 |  |
|  |  |  |  | Lowakami/lengitsandzana naye akanginikanga imvume kucalisa umntfwana ema ARVs | | | 12 |  |
|  |  |  |  | Umngani/sihlobo sami satsi ngingamniki umntfwana ema ARV | | | 13 |  |
|  |  |  |  | **Stigma/disclosure** | | | |  |
|  |  |  |  | Ngite indzawo lefihlakele lapho ngingabeka khona imitsi yami | | | 14 |  |
|  |  |  |  | Encenye singa daluleka simo sami sengati | | | 15 |  |
|  |  |  |  | Encenye singa daluleka simo sami sengati seluswane lwami | | | 16 |  |
|  |  |  |  | Bengingafuni kubatjela ekhaya kutsi luswane lwami lutsatsa ema ARV | | | 17 |  |
|  |  |  |  | Bengingafuni kutjela wakami kutsi luswane lwami lutsatsa ema ARV | | | 18 |  |
|  |  |  |  | **Clinic** | | | |  |
|  |  |  |  | Ekliniki bekute imitsi leyenele | | | 19 |  |
|  |  |  |  | Abaphatsani kahle | | | 20 |  |
|  |  |  |  | Lusito lwakhona alusilo lolukahle | | | 21 |  |
|  |  |  |  | Kumiwa sikhatsi lesidze ekliniki | | | 22 |  |
|  |  |  |  | Bebangasitani kumake neluswane kanye kanye ekliniki | | | 23 |  |
|  |  |  |  | Ekliniki bebanganiketi lolusito luswane/nami besiludzinga (e.g. kudla) chaza ____________ | | | 24 |  |
|  |  |  |  | **Information** | | | |  |
|  |  |  |  | Ekliniki batsite luswane lusengakalungeli kunatsa ema ARV | | | 25 |  |
|  |  |  |  | **Medication** | | | |  |
|  |  |  |  | Ekliniki labanginika yona luswane/nami yaba nemitselela lemibi. | | | 26 |  |
|  |  |  |  | Lomutsi awusebenti | | | 27 |  |
|  |  |  |  | Nginconota kunika luswane lwami imitsi yesintfu | | | 28 |  |
|  |  |  |  | Yangilahlekela imitsi yeluswane lwami | | | 29 |  |
|  |  |  |  | Bayeba imitsi yeluswane lwami | | | 30 |  |
|  |  |  |  | Ngayicedza imitsi yeluswane lwami | | | 31 |  |
|  |  |  |  | **Lokunye** | | | |  |
|  |  |  |  | Lokunye 1:_____________________ | | | 32 |  |
|  |  |  |  | Lokunye 2:_____________________ | | | 33 |  |
|  |  |  |  | Lokunye 3:_____________________ | | | 34 |  |
|  |  |  |  | Lokunye 4: ____________________ | | | 35 |  |
|  |  |  |  | Lokunye 5: ____________________ | | | 36 |  |
|  |  |  |  | *Angati* | | | *88* |  |
|  |  |  |  | *Kwala* | | | *99* |  |
| **C17** | Luswane lwakho lugcine nini kutsatsa ema ARV?  *If participant answers “don’t know”, probe to nearest month. If she refuses to answer, enter 99.* | | | Ngaphansi kwelilanga | | | 1 |  |
|  |  |  |  | Ngaphansi kweliviki | | | 2 |  |
|  |  |  |  | Ngaphansi kwenyanga | | | 3 |  |
|  |  |  |  | Emkhatsini wenyanga netinyanga letintsatfu | | | 4 |  |
|  |  |  |  | Ngetulu kwetinyanga letintsatfu | | | 5 |  |
|  |  |  |  | *Angati* | | | *88* |  |
|  |  |  |  | *Kwala* | | | *99* |  |

| **SeCTION D. PATIENT CARD ABSTRACTION** | | | | | |
| --- | --- | --- | --- | --- | --- |
| **No.** | **QUESTIONS & INSTRUCTIONS** | | **RESPONSES** | | **skips** |
| *Interviewer should only review information from maternal HIV card if mom is LTF, and information from infant CWC card if infant is LTF, i.e., if patient is not LTF, then information should not be reviewed.*  **LOBUTAKO:** Nyalo ke, ngicela kubuta kutsi ngingalibona yini likhadi lakho lekunakekela nge HIV (green card) neye kunakekelwa ye luswane I Child Welfare Card. Ngicela kutsatsa lamalanga lowaya ngawo emtfolamphilo (nelweluswane uma kungenteka) lalapha ekhadini, naleminye imininingwane yetemphilo. Ungatsandza yini kutsi ungibonise kona. | | | | | |
| **D01** | [DO NOT ASK IF INFANT DIED >6 WKS AFTER DELIVERY from T05]  Do you have your infant’s Child Welfare Card? | | Yes | 1 |  |
|  |  |  | No | 2 |  |
|  |  |  | *Refusal* | *99* |  |
|  |  |  | *Not applicable* | 77 |  |
| **D02** | Infant DOB: | | \|  \|  \|  \|  \|  \|  \|  \|  \|  \|  \| \| --- \| --- \| --- \| --- \| --- \| --- \| --- \| --- \| --- \| --- \| \| **Day** \| \|  \| **Month** \| \|  \| **Year** \| \| \| \| \| | | |
| **D03** | Infant Vaccinations: | **Visit Type** | | **Immunization Date** |  |
|  |  | **At birth** | |  |  |
|  |  | BCG | | / / |  |
|  |  | OPV 0 | | / / |  |
|  |  | **6 weeks** | |  |  |
|  |  | OPV 1 | | / / |  |
|  |  | DPT/HepB/Hib 1 | | / / |  |
|  |  | PCV 1 | | / / |  |
|  |  | **10 weeks** | |  |  |
|  |  | OPV 2 | | / / |  |
|  |  | DPT/HepB/Hib 2 | | / / |  |
|  |  | PCV 2 | | / / |  |
|  |  | **14 weeks** | |  |  |
|  |  | OPV 3 | | / / |  |
|  |  | DPT/HepB/Hib 3 | | / / |  |
|  |  | **9 months** | |  |  |
|  |  | Measles 1 | | / / |  |
|  |  | PCV 3 | | / / |  |
|  |  | **18 months** | |  |  |
|  |  | OPV 4 | | / / |  |
|  |  | Measles 2 | | / / |  |
|  |  | OPV 5 | | / / |  |
| **D04** | Infant Visits: | **Visit Type** | **Visit Date** | **DBS Barcode** | **DBS Result (R/NR/U)** |
|  |  | **0-7 days** |  |  |  |
|  |  | **7-14 days** |  |  |  |
|  |  | **6 weeks** |  |  |  |
|  |  | **10 weeks** |  |  |  |
|  |  | **14 weeks** |  |  |  |
|  |  | **6 months** |  |  |  |
|  |  | **9 months** |  |  |  |
|  |  | **12 months** |  |  |  |
|  |  | **15 months** |  |  |  |
|  |  | **18 months** |  |  |  |
|  |  | **24 months** |  |  |  |
| **D05** | Do you have your HIV Care Card (green card)? | Yes | | 1 |  |
|  |  | No | | 2 |  |
|  |  | *Refusal* | | *99* |  |
|  |  | *Not applicable* | | 77 |  |
| **D06** | Maternal HIV care visits: | **Visit Date** | | **ART Prescribed?** *Indicate ‘Y’ for ‘yes’ or ‘N’ for ‘no’.* | |
|  |  | / / | |  | |
|  |  | / / | |  | |
|  |  | / / | |  | |
|  |  | / / | |  | |
|  |  | / / | |  | |
|  |  | / / | |  | |
|  |  | / / | |  | |
|  |  | / / | |  | |
|  |  | / / | |  | |
|  |  | / / | |  | |
|  |  | / / | |  | |
|  |  | / / | |  | |
|  |  | / / | |  | |
|  |  | / / | |  | |
|  |  | / / | |  | |
|  |  | / / | |  | |
|  |  | / / | |  | |
|  |  | / / | |  | |
|  |  | / / | |  | |
|  |  | / / | |  | |
|  |  | / / | |  | |
|  |  | / / | |  | |

| **T09** | Interview stop time: | \|  \|  \| **:** \|  \|  \| \| --- \| --- \| --- \| --- \| --- \| \| **Hour** \| \|  \| **Minute** \| \| |
| --- | --- | --- | --- | --- | --- | --- | --- | --- | --- | --- | --- | --- |

| **Feedback** |
| --- |
| **QUESTIONS & INSTRUCTIONS** |
| Please provide any other comments:  ***____________________________________________________________________________________________________________________________________________________________________________________________________________________________________________________________________________________________________________________________________________________________________________________________________________________________________________________________________________________________________________________________________________________________________________________________________________*** |
